# A Peptide-Nucleic Acid Targeting miR-335-5p Enhances Expression of Cystic Fibrosis Transmembrane Conductance Regulator (*CFTR*) Gene with the Possible Involvement of the CFTR Scaffolding Protein NHERF1

**DOI:** 10.3390/biomedicines9020117

**Published:** 2021-01-26

**Authors:** Anna Tamanini, Enrica Fabbri, Tiziana Jakova, Jessica Gasparello, Alex Manicardi, Roberto Corradini, Alessia Finotti, Monica Borgatti, Ilaria Lampronti, Silvia Munari, Maria Cristina Dechecchi, Giulio Cabrini, Roberto Gambari

**Affiliations:** 1 Section of Molecular Pathology, Department of Pathology and Diagnostics, University-Hospital of Verona, 37126 Verona, Italy; anna.tamanini@aovr.veneto.it (A.T.); silvia.munari88@gmail.com (S.M.); 2 Department of Life Sciences and Biotechnology, University of Ferrara, 44121 Ferrara, Italy; enrica.fabbri@unife.it (E.F.); jessica.gasparello@unife.it (J.G.); alessia.finotti@unife.it (A.F.); monica.borgatti@unife.it (M.B.); ilaria.lampronti@unife.it (I.L.); 3 Department of Chemistry, Life Sciences and Environmental Sustainability, University of Parma, 43124 Parma, Italy; tiziana.jakova@studenti.unipr.it (T.J.); alex.manicardi@unipr.it (A.M.); roberto.corradini@unipr.it (R.C.); 4 Research Center on Innovative Therapies for Cystic Fibrosis, University of Ferrara, 44121 Ferrara, Italy; giulio.cabrini@unife.it; 5 Department of Neurosciences, Biomedicine and Movement, University of Verona, 37100 Verona, Italy; mcristina.dechecchi@gmail.com

**Keywords:** peptide nucleic acids, cystic fibrosis, microRNAs, miR-335-5p, miRNA targeting, delivery, NHERF1, CFTR

## Abstract

(1) Background: Up-regulation of the Cystic Fibrosis Transmembrane Conductance Regulator gene (*CFTR*) might be of great relevance for the development of therapeutic protocols for cystic fibrosis (CF). MicroRNAs are deeply involved in the regulation of CFTR and scaffolding proteins (such as NHERF1, NHERF2 and Ezrin). (2) Methods: Content of miRNAs and mRNAs was analyzed by RT-qPCR, while the CFTR and NHERF1 production was analyzed by Western blotting. (3) Results: The results here described show that the CFTR scaffolding protein NHERF1 can be up-regulated in bronchial epithelial Calu-3 cells by a peptide-nucleic acid (PNA) targeting miR-335-5p, predicted to bind to the 3′-UTR sequence of the *NHERF1* mRNA. Treatment of Calu-3 cells with this PNA (R8-PNA-a335) causes also up-regulation of CFTR. (4) Conclusions: We propose miR-335-5p targeting as a strategy to increase CFTR. While the efficiency of PNA-based targeting of miR-335-5p should be verified as a therapeutic strategy in CF caused by stop-codon mutation of the *CFTR* gene, this approach might give appreciable results in CF cells carrying other mutations impairing the processing or stability of CFTR protein, supporting its application in personalized therapy for precision medicine.

## 1. Introduction

MicroRNAs (miRNAs) are noncoding RNAs from 19 to 25 nucleotides in length that regulate gene expression by targeting mRNAs, leading to a translational repression or mRNA degradation [1,2,3]. The complex networks constituted by miRNAs and mRNA targets lead to the control of highly regulated biological functions, such as differentiation, cell cycle and apoptosis [4,5,6,7]. With respect to cystic fibrosis (CF), miRNA-based post-transcriptional regulation of the expression of the Cystic Fibrosis Transmembrane Conductance Regulator (*CFTR*) gene and CFTR-regulators has recently been explored by different groups in CF primary bronchial epithelial cells in vitro or from bronchial brushings ex vivo [7,8,9,10,11]. Gillen et al. [7] reported that the expression of miR-145 and miR-494 is anti-regulated in respect to CFTR expression. Genome-wide studies of miRNAs in primary non-CF bronchial epithelial cells demonstrated that miR-138 is a down-regulator of SIN3A, a transcriptional repressor of *CFTR* gene [9]. A second analysis of miRNA profiling showed high expression of miR-494 and miR-509-3p in CF cells and a direct interaction with the *CFTR* transcript [10]. These in vitro findings were confirmed and extended in ex vivo analyses which evidenced an increased expression of miR-494, miR-223 and miR-145 in CF brushings of airway cells [11]. Altogether, different miRNAs which have been found to be increased in CF primary bronchial epithelial cells can reduce CFTR expression, either by direct (miR-145, miR-223, miR-494, miR-509-3p) or indirect (miR-138) targeting.

In this respect, it should be underlined that in addition to direct interaction with *CFTR* mRNA, miRNAs might regulate CFTR, at least in theory, through binding to 3′-UTR of mRNAs coding CFTR regulators, such as a large series of proteins known to interact with the CFTR protein in the quality control mechanisms to modulate its folding and misfolding [12]. Among the so-termed “CFTR interactome”, some proteins have been shown to interact with CFTR to stabilize its expression on the apical membrane of the epithelial cells [13,14]. The CFTR carboxyl terminal tail, which possesses a PDZ protein-binding motif in the last four amino acids of the CFTR protein (D1477-TRL1480), has been found to interact with a number of scaffolding proteins that are primarily localized at the apical surfaces of epithelial cells, that include, but are not limited to, NHERF1, NHERF2, PDZK1, PDZK2 and Shank2. NHERF1 functionally stabilizes cell-surface CFTR. Indeed, it has been observed that NHERF1 overexpression induces the redistribution of CFTR from the cytoplasm to the plasma membrane and rescued mutant F508del-CFTR activity via the re-organization of the actin cytoskeleton by inducing the formation of the NHERF1-RhoA-ROCK-Ezrin-actin multiprotein complex [15,16,17,18].

In the field of therapeutic molecules, peptide nucleic acids (PNAs) have been recently reported to be of great interest. PNAs are DNA analogues of outstanding properties [19,20,21,22,23] since, despite a radical structural change with respect to DNA and RNA, they are capable of sequence-specific and efficient hybridization with complementary DNA and RNA, forming Watson–Crick double helices [19,20]. In addition, they are able to generate triple helix formation with double stranded DNA and perform strand invasion [21,22]. Accordingly, they have been used as very efficient tools for pharmacologically mediated alteration of gene expression, both in vitro and in vivo [24,25,26,27,28]. PNAs and PNA-based analogues were proposed as antisense molecules targeting mRNAs, triple-helix forming molecules targeting eukaryotic gene promoters, artificial promoters, decoy molecules targeting transcription factors [27,28]. Recently, PNAs have been shown to be capable of altering biological functions of microRNAs, both in vitro and in vivo [28,29,30,31,32,33,34,35].

With respect to PNA-based targeting of miRNAs involved in *CFTR* regulation, we recently reported that a PNA directed against miR-145-5p inhibits its biological functions and up-regulates CFTR expression [36].

The goal of this study was to verify whether down-regulation of miRNAs targeting NHERF1 might induce effects on NHERF1 expression, supporting possible indirect effects on CFTR expression and functional correction. The selected miRNA was miR-335-5p [37] for the following reasons: (a) NHERF1 is a putative miR-335-5p target [38]; (b) potential miR-335-5p binding sites are described in the 3′-UTR of *CFTR* mRNA [38]; (c) miR-335-5p is expressed in the lung [37]. Specific down-regulation of miR-335-5p was achieved with a PNA carrying a R8 poly-arginine motif to facilitate its uptake by target cells. We then verified whether this was associated with increased expression of NHERF1 and CFTR.

## 2. Experimental Section

### 2.1. Synthesis and Characterization of PNAs

Synthesis and characterization of anti-miRNA PNAs were similar to those previously reported [33]. PNA-a101 was synthesized as previously reported [34]. The synthesis of PNA-a335, PNA-a335-MUT, PNA-a96 and PNA-a183 was performed using standard Fmoc-based automate peptide synthesizer (Syro I, MultiSynTech GmbH, Witten, Germany), using a ChemMatrix-RinkAmide resin loaded with Fmoc-Gly-OH (0.2 mmol/g) as first monomer and using commercially available monomers (Link Technologies, Bellshill, UK) with HBTU/DIPEA coupling. Cleavage from the solid support was performed with 10% m-cresol in trifluoroacetic acid, followed by precipitation and washings with diethyl ether. Purification was performed by HPLC using a Phenomenex Jupiter RPC18, (250 × 4.6 mm, 1.7 μm) column. Gradient: 100% A for 5 min, then from 0% to 50% B in 30 min at 4 mL/min flow (A: water + 0.1% trifluoroacetic acid; B: acetonitrile + 0.1% trifluoroacetic acid). After purification, the PNA’s identity and purity were confirmed by UPLC/ESI-MS (Waters Acquity ultra performance LC HO6UPS-823M, with Waters SQ detector equipped with Waters UPLC BEH C18, 50 × 2.1 mm, 1.7 μm) at 35 °C (Supplementary Figures S1–S5). The concentration of the PNA was determined using UV-absorbance at 260 nm assuming an additive contribution of nucleobases.

*R8-PNA-a335:* sequence H-R_8_-TTTCGTTATTGCTCTTGA-Gly-NH_2_; UPLC/ESI-MS Rt = 2.66 min, calculated MW: 6164.30 g/mol; m/z found (calculated): 1233.8 (1233.86) [MH_5_]^5+^, 1028.2 (1028.38) [MH_6_]^6+^, 881.5 (881.61) [MH_7_]^7+^, 771.4 (771.53) [MH_8_]^8+^, 685.8 (685.92) [MH_9_]^9+^.

*R8-PNA-a335-MUT:* sequence H-R_8_-TTTAGTTCTTGCGCTTTA-Gly-NH_2_ (mutated bases underlined); UPLC/ESI-MS Rt = 2.67 min, calculated MW: 6164.30 g/mol; m/z found (calculated): 1233.7 (1233.86) [MH_5_]^5+^, 1028.4 (1028.38) [MH_6_]^6+^, 881.6 (881.61) [MH_7_]^7+^, 771.6 (771.53) [MH_8_]^8+^, 686.0 (685.92) [MH_9_]^9+^.

*R8-PNA-a96:* sequence H-R_8_-AAATGTGCTAGTGCCAAA-Gly-NH_2_; UPLC/ESI-MS Rt = 2.61 min, calculated MW: 6234.36 g/mol; m/z found (calculated): 1247.8 (1247.87) [MH_5_]^5+^, 1039.9 (1040.06) [MH_6_]^6+^, 891.7 (891.62) [MH_7_]^7+^, 780.3 (780.30) [MH_8_]^8+^, 693.8 (693.70) [MH_9_]^9+^, 624.5 (624.44) [MH_10_]^10+^.

*R8-PNA-a183:* sequence H-R_8_-AATTCTACCAGTGCCATA-Gly-NH_2_; UPLC/ESI-MS Rt = 2.52 min, calculated MW: 6145.31 g/mol; m/z found (calculated): 1230.2 (1230.06) [MH_5_]^5+^, 1025.2 (1025.22) [MH_6_]^6+^, 879.0 (878.90) [MH_7_]^7+^, 769.2 (769.16) [MH_8_]^8+^, 683.7 (683.81) [MH_9_]^9+^, 615.5 (615.53) [MH_10_]^10+^,559.7 (559.67) [MH_11_]^11+^, 513.1 (513.11) [MH_12_]^12+^.

### 2.2. Cell Lines and Culture Conditions

The bronchial epithelial Calu-3 cells [39] were cultured in humidified atmosphere of 5% CO_2_/air in DMEM/F12 medium (Gibco, Grand Island, NY, USA) supplemented with 10% fetal bovine serum (Biowest, Nauillè, Francia), 100 units/mL penicillin and 100 µg/mL streptomycin (Lonza, Verviers, Belgio) and 1% NEEA (100×) (non-essential amino acids solution) (Gibco, Grand Island, NY, USA). Fischer rat thyroid (FRT) epithelial cells, stably co-expressing human F508del CFTR and the high-sensitivity halide-sensing green fluorescent analog yellow fluorescent protein (HS-YFP) YFP-H148Q/I152L (FRT-F508del) were a generous gift from Dr. L. J. Galietta (Telethon Institute of Genetics and Medicine, Pozzuoli, Italy) [40,41]. FRT-F508del cells were grown in Coon’s modified Ham’s F-12 medium plus 10% FBS, L-glutamine, and penicillin/streptomycin at 37 °C under 5% CO_2_. To determine the effect on proliferation, cell growth was monitored by determining the cell number/mL using a Z2 Coulter Counter (Coulter Electronics, Hialeah, FL, USA). The CFTR corrector VX809 was obtained from Selleckchem (Houston, TX, USA).

### 2.3. RNA Extraction

Cultured cells were trypsinized and collected by centrifugation at 1500 rpm for 10 min at 4 °C, washed with PBS, and lysed with Tri-Reagent (Sigma Aldrich, St. Louis, MO, USA), according to the manufacturer’s instructions. The isolated RNA was washed once with cold 75% ethanol, dried and dissolved in nuclease free pure water before use.

### 2.4. Quantitative Analyses of miRNAs

For miRNA quantification using real-time RT-qPCR reagents, the primers and probes (hsa-miR-335-5p assay ID: 000546; hsa-miR-101-3p assay ID: 002253) were obtained from Applied Biosystems (Applied Biosystems, Foster City, CA, USA). Reverse transcriptase (RT) reactions were performed using the TaqMan MicroRNA Reverse Transcription Kit (Thermo-Fisher Scientific, Waltham, MA, USA); real-time PCR was performed according to the manufacturer’s protocols [42]. The quantity of 300 ng of total RNA was used for each RT reaction. All RT reactions, including no-template controls and RT-minus controls, were quantified in duplicate using the CFX96 Touch Real-Time PCR Detection System (BioRad, Hercules, CA, USA). The relative expression was calculated using the comparative cycle threshold method and U6 snRNA (assay ID: 001973) was used, as reference, to normalize all RNA samples, since it remains constant in the assayed samples by miR-profiling and quantitative RT-PCR analysis, as previously reported [31,32,33,34].

### 2.5. Analysis of NHERF1 and CFTR Expression: RT-qPCR

Gene expression analysis was performed by RT-qPCR using 300 ng of total RNA, extracted and reverse transcribed using random hexamers as RT reaction primers. Quantitative real-time PCR (RT-qPCR) assays were carried out using gene-specific double fluorescently labeled probes. Primers and probes used to assay CFTR (Assay ID: Hs00357011_m1) and NHERF1 (also known as SLC9A3R1, Assay ID: Hs00188594_m1) gene expression were purchased from Applied Biosystems. The relative expression was calculated using the comparative cycle threshold method and, as reference genes, the human RPL13A (Assay ID: Hs03043885_g1).

### 2.6. Analysis of NHERF1 and CFTR Expression: Western Blotting 

CFTR and NHERF1 expression was measured in Western blotting. Cell pellets were lysed in 1% Nonidet P40 (IGEPAL), 0.5% sodiumdeoxycholate, 200 mM NaCl, 10 mM Trizma base, pH 7.8, 1 mM EDTA plus protease inhibitor mixture and 1 mM PMSF for 30 min on ice. Lysates were cleared by centrifugation at 10,000× *g* for 10 min at 4 °C. Protein concentration was determined by the method of Lowry after precipitation with 5% Trichloroacetic acid (TCA), utilizing bovine serum albumin (BSA) as a standard. For CFTR analysis, 20 μg of the total proteic extracts were heated in Laemmli buffer (Bio-Rad, Hercules, CA, USA) at 37 °C for 10 min and loaded onto a 3 to 8% Tris-acetate gel (Bio-Rad, Hercules, CA, USA). The gel proteins were transferred to polyvinylidene difluoride (PVDF) membrane (Bio-Rad, Hercules, CA, USA) by using Trans Blot Turbo (Bio-Rad Laboratories) and processed for Western blotting by using mouse monoclonal antibody, clone 596, against NBD2 domain of CFTR (University of North Carolina, Cystic Fibrosis Center, Chapel Hill, NC, USA) at a dilution of 1:2500 with an overnight incubation at 4 °C. For NHERF1 blotting, 20 μg of the total extracts were heated in Laemmli buffer (Bio-Rad, Hercules, CA, USA) at 95 °C for 3 min. The lysates were separated on 4–15% SDS polyacrylamide gels (Bio-Rad, Hercules, CA, USA). The gel proteins were transferred to PVDF membrane (Bio-Rad, Hercules, CA, USA). Filters were incubated with a monoclonal antibody to NHERF1 at a dilution of 1:1000 (BD Franklin Lakes, NJ, USA) overnight at 4 °C. After washes, membranes were incubated with horseradish peroxidase-coupled anti-mouse immunoglobulin (R&D System, Minneapolis, MN, USA) at room temperature for 1 h and after washes the signal was developed by enhanced chemiluminescence (LumiGlo Reagent and Peroxide, Cell Signaling, Danvers, MA, USA). After membrane stripping, β-Actin monoclonal antibody (Sigma Aldrich, St. Louis, MO, USA) was used to investigate the equal loading of samples [43,44].

### 2.7. Analysis of the CFTR Function

FRT cells co-expressing human F508del CFTR and yellow fluorescent protein (YFP H148Q/I152L), grown on round glass coverslips, were treated for 30 min with forskolin (20 µM) and genistein (50 µM) before the fluorescence assay. CFTR-dependent chloride efflux was analyzed by single-cell fluorescence imaging of YFP fluorescence quenching by iodide, stimulated by forskolin and genistein, in the presence and in the absence of CFTR Inh-172 (10 µM), as described in [45,46].

### 2.8. Analysis of Apoptosis

Annexin V and Dead Cell assays on Calu-3 cells, untreated and treated for 72 h with 4 µM PNA-a335, 5 µM Stattic together with 10% DMSO, were performed with the Muse^®^ Cell Analyzer (Merck Millipore, Burlington, MA, USA) method, according to the instructions supplied by the manufacturer. This procedure utilizes Annexin V to detect phosphatidyl serine (PS) on the external membrane of apoptotic cells. A dead cell marker, 7-aminoactinomycin D (7-ADD), is also used as an indicator of cell membrane structural integrity. 7-ADD is excluded from live, healthy cells, as well as early apoptotic cells. Cells were washed with sterile PBS 1X, trypsinized, suspended and diluted (1:2) with the one step addition of the Muse Annexin V and Dead Cell reagent. After incubation for 10 min at room temperature in the dark, samples were put on ice, vortexed and analyzed. Data from prepared samples were acquired and recorded utilizing the Annexin V and Dead Cell Software Module (Merck Millipore, Burlington, MA, USA) [33,47].

### 2.9. Next Generation Sequencing (RNA-Seq)

Next generation sequencing (NGS) experiments were performed at the Laboratory for Technologies of Advanced Therapies (LTTA) of Ferrara University. SmallRNA libraries were prepared from total RNA using TruSeq^®^ Small RNA Library PrepKit v2 (Illumina, RS-200-0012/24/36/48, San Diego, CA, USA), according to manufacturer’s indications. Briefly, 35 ng of purified RNA were linked to RNA 3′ and 5′ adapters, converted in cDNA and amplified using Illumina primers containing unique indexes for each sample. Libraries were quantified through an Agilent Bioanalyzer, using the High Sensitivity DNA kit (Agilent, 5067-4626, Santa Clara, CA, USA), and equal amounts of libraries were pooled together and submitted to size-selection in order to keep only fragments between 130 and 160 bp. After ethanol precipitation, the library pool was quantified through the Agilent Bioanalyzer and High Sensitivity DNA kit, denatured and diluted to 1.8 pM and sequenced using Illumina NextSeq500 platform and NextSeq^®^ 500/550 High Output Kit v2 (75 cycles) (Illumina, FC-404-2005, San Diego, CA, USA). Raw base-call data generated from the Illumina NextSeq500 system were demultiplexed automatically by the Illumina BaseSpace Sequence Hub (https://basespace.illumina.com/home/index) and converted to FASTQ format. After a quality check, using the FastQC tool (https://www.bioinformatics.babraham.ac.uk/projects/fastqc/), adapter sequences were trimmed by Cutadapt (http://cutadapt.readthedocs.io/en/stable/index.html). In this step, sequences shorter than 10 nucleotides were also removed. Read mapping was performed using the STAR algorithm (https://www.ncbi.nlm.nih.gov/pubmed/23104886), and the reference genome was composed of human microRNAs sequences from the miRbase [5] (http://www.mirbase.org/). Counting of raw mapped reads was performed using the htseq-count script from the HTSeq tools (http://www-huber.embl.de/HTSeq/doc/overview.html); raw counts were normalized using the DESeq2 bioconductor package (http://bioconductor.org/packages/release/bioc/html/DESeq2.html).

### 2.10. Statistical Analysis

Results are expressed as mean ± standard error of the mean (SEM). Comparisons between groups were made by using one-way ANOVA (* *p* < 0.05, ** *p* < 0.01, *** *p* < 0.001 and **** *p* < 0.0001).

## 3. Results

### 3.1. Presence of miR-335-5p Binding Sites within NHERF1 3′-UTR mRNA Sequences

**Figure 1:**
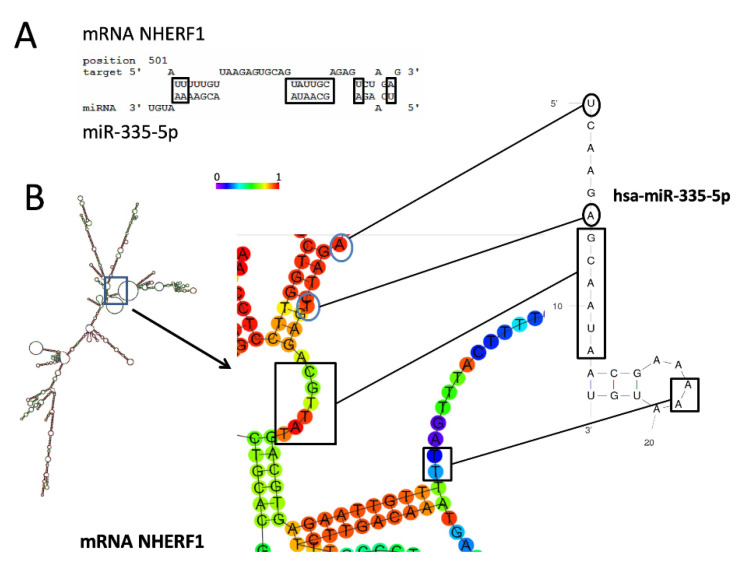
Computer-aided analysis of the possible pairing interaction between hsa-miR-335-5p (entry: http://www.mirbase.org/cgi-bin/mirna_entry.pl?acc=MI0000816) and *NHERF1* mRNA (entry: https://www.ncbi.nlm.nih.gov/nuccore/381214354). The RNAhybrid tool for the prediction of miRNA and mRNA interaction (https://bibiserv2.cebitec.uni-bielefeld.de/rnahybrid) [47], the UNAfold web server (http://unafold.rna.albany.edu/), and the RNAfold WebServer (http://rna.tbi.univie.ac.at/cgi-bin/RNAWebSuite/RNAfold.cgi) were used. (**A**) Complementarity between miR-335-5p and *NHERF1* mRNA miR-335 site; (**B**) magnification of the stem-loop secondary structure indicating possible interactions. Colors in the schematic structure refer to probability of base pairing or unpairing (remaining as single base). Red segments contain the paired (or unpaired) bases of the highest probability.

Figure 1A shows the location of the putative miR-335-5p binding site within the *NHERF1* 3′-UTR mRNA sequence, as found employing RNAhybrid, a tool for finding the minimum free energy hybridization of a short sequence RNA to the best fitting part of a long one. This tool is mainly considered for microRNA target prediction (https://bibiserv2.cebitec.uni-bielefeld.de/rnahybrid). Moreover, miR-335-5p targeting *NHERF1* can be also considered after an inspection of recently published papers [48]. The extent of the interaction between the *NHERF1* mRNA 3′-UTR (https://www.ncbi.nlm.nih.gov/nuccore/381214354) and mature miR-335-5p involves 10 nucleotides (boxed in Figure 1A), located starting from nucleotide position 501 of the *NHERF1* 3′-UTR and spatially described in Figure 1B [49].

### 3.2. MicroRNA miR-335-5p Exhibits High Complementarity with the 3′-UTR Sequence of NHERF1 mRNA 

**Figure 2:**
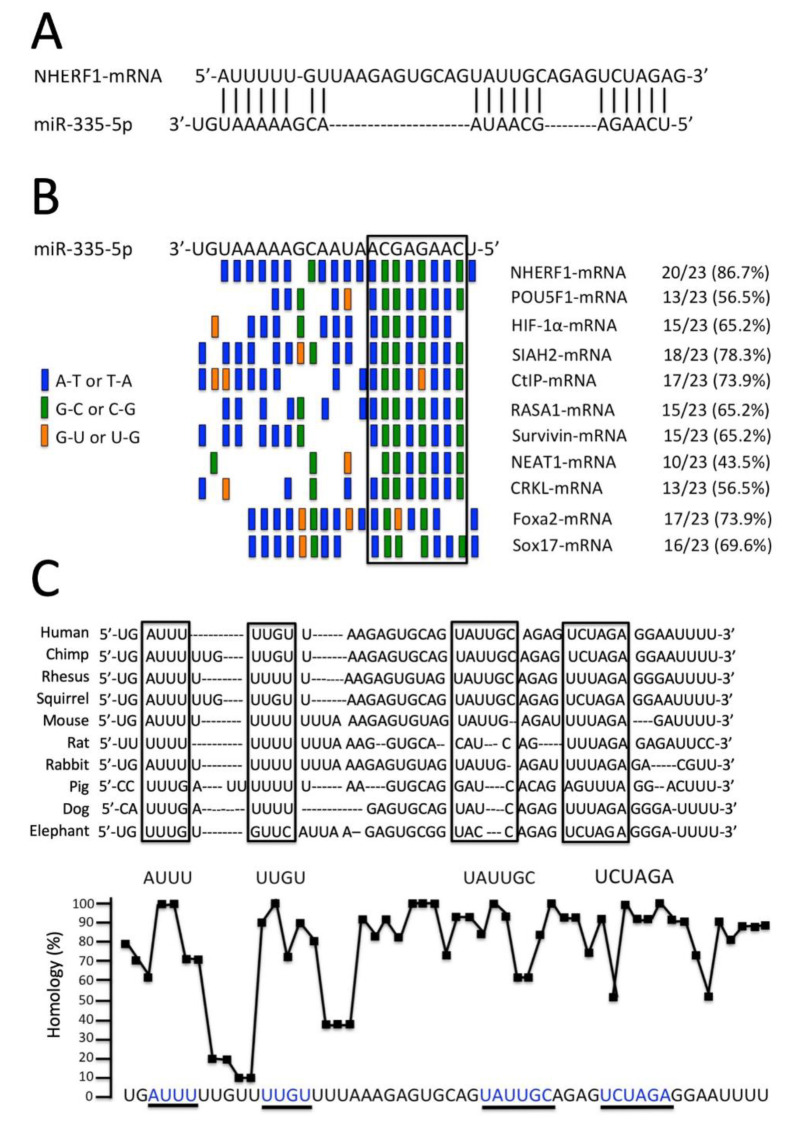
(**A**) Predicted interaction between miR-335-5p and 3′-UTR of *NHERF1* mRNA. (**B**) Comparison of NHERF1 binding to miR-335-5p to that of other validated miR-335-5p regulated mRNAs. This is, to our knowledge, the highest value described in the literature. In fact, lower values were found (Figure 2B) when the analysis was conducted on the interactions between miR-335-5p sequence and validated miR-335-5p regulated mRNAs. POU5F1: POU Class 5 Homeobox 1 [50]; HIF-1α: Hypoxia-inducible factor 1-alpha [51]; SIAH2: Siah E3 Ubiquitin Protein Ligase 2 [52]; CtIP: CTBP-Interacting Protein [53]; RASA1: RAS P21 Protein Activator 1 [54]; survivin [55]; NEAT1: Nuclear Paraspeckle Assembly Transcript 1 [56]; CRKL: CRK Like Proto-Oncogene, Adaptor Protein [57]; Foxa2: Forkhead Box A2 [58] and Sox17: SRY-Box 17 [58]. (**C**) The binding sites for miR-335-5p are conserved throughout molecular evolution of the *NHERF1* gene. Upper part of the panel: alignment of the miR-335-5p binding sites within the 3′-UTR region of *NHERF1* gene of *Homo sapiens* (Human), *Pan troglodytes* (Chimp), *Macaca Mulatta* (Rhesus), *Saimiri sciureus* (Squirrel), *Mus musculus* (Mouse), *Rattus norvegicus* (Rat), *Oryctolagus cuniculus* (Rabbit), *Sus scrofa* (Pig), *Bos taurus* (Cow), *Felis catus* (Cat), *Canis lupus familiaris* (Dog), *Myotis lucifugus* (Brown bat) and *Loxodonta africana* (Elephant). Lower part of the panel: level of homology shown for each nucleotide. The sequences complementary to miR-335-5p (see panel A) are boxed and underlined in the upper and lower part of the panel, respectively.

Figure 2A shows the base-pairing interaction between miR-335-5p and the target sequences present within the 740 bp long 3′-UTR of the *NHERF1* mRNA. This interaction is characterized by 15 AU/UA and 5 CG/GC bonds. In total, the molecular interaction between miR-335-5p and *NHERF1* mRNA involves 20/23 base pairing for a total of 86.7% of the miR-335-5p mature sequence. This is, to our knowledge, the highest value described in the literature. In fact, lower values were found (Figure 2B) when the analysis was conducted on the interactions between the miR-335-5p sequence and validated miR-335-5p regulated mRNAs such as those coding POU Class 5 Homeobox 1 (POU5F1) [50], Hypoxia-inducible factor 1-alpha (HIF-1α) [51], Siah E3 Ubiquitin Protein Ligase 2 (SIAH2) [52], CTBP-Interacting Protein (CtIP) [53], RAS P21 Protein Activator 1 (RASA1) [54], survivin [55], Nuclear Paraspeckle Assembly Transcript 1 (NEAT1) [56], CRK Like Proto-Oncogene, Adaptor Protein (CRKL) [57], Forkhead Box A2 (Foxa2) and SRY-Box 17 (Sox17) [58]. This strongly suggests that the 3′-UTR sequence of *NHERF1* mRNA is an authentic molecular target of miR-335-5p, since its complementarity to the 3′-UTR of *NHERF1* mRNA is much higher than that found in several validated miR-335-5p regulated mRNAs so far described (Figure 2B).

### 3.3. The miR-335-5p Binding Sites of the 3′-UTR Sequence of NHERF1 mRNA Are Conserved throughout Evolution

The possibility that the miR-335-5p binding sequence of NHERF1 is highly conserved through evolution would suggest a role of miR-335-5p in NHERF1 expression and functions. In Figure 2C, the alignment is shown of the miR-335-5p binding sites of *Homo sapiens* (Human), *Pan troglodytes* (Chimp), *Macaca Mulatta* (Rhesus), *Saimiri sciureus* (Squirrel), *Mus musculus* (Mouse), *Rattus norvegicus* (Rat), *Oryctolagus cuniculus* (Rabbit), *Sus scrofa* (Pig), *Bos taurus* (Cow), *Felis catus* (Cat), *Canis lupus familiaris* (Dog), *Myotis lucifugus* (Brown bat) and *Loxodonta africana* (Elephant). This analysis underlines differences with respect to the *Homo sapiens* sequence. The NHERF1 sequences complementary to miR-335-5p (see Figure 2A) are boxed. These regions show the highest level of homology (reaching 100% homology in some positions), as indicated in the bottom part of Figure 2C, where these regions are underlined, and the analysis of the homology is shown for each nucleotide. Taken together, the data shown in Figure 2 sustain the concept of a role of miR-335-5p binding sites in *NHERF1* gene regulation.

### 3.4. R8-PNA-a335 Inhibits miR-335-5p

**Figure 3:**
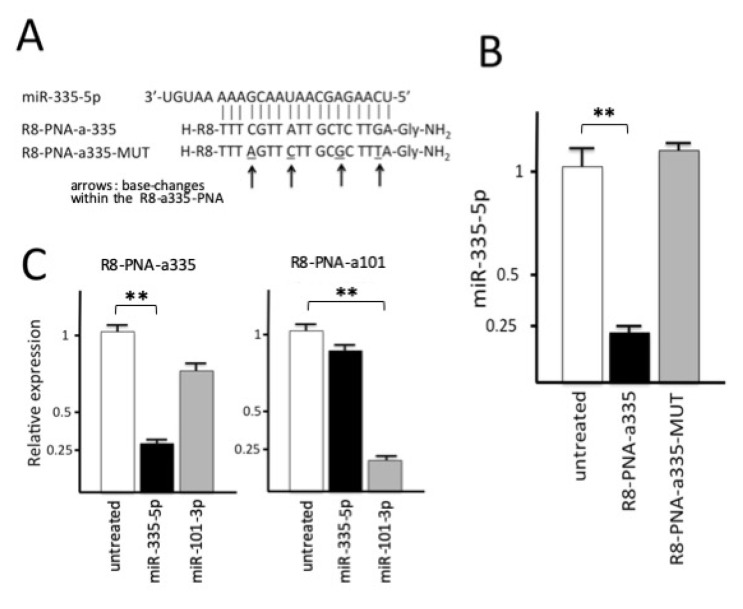
(**A**) Sequences of the R8-PNA-a335 and of the mutated R8-PNA-a335-MUT. The mutations in the R8-PNA-a335-MUT are underlined and arrowed. (**B**) Inhibition of miR-335-5p hybridization signals in Calu-3 cells treated for 72 h with R8-PNA-a335 (black box) and mutated R8-PNA-a335-MUT (gray box) molecules (4 µM); white boxes: untreated Calu-3 cells. (**C**) Effects of treatments of Calu-3 cells with R8-PNA-a335 and R8-PNA-a101 on miR-335-5p (black boxes) and miR-101-3p (gray boxes) hybridization signals. Treatments were carried out for 72 h with 4 µM R8-PNA-a335 and R8-PNA-a101. Results are expressed as mean ± standard error of the mean (SEM) of at least three independent experiments. ** Statistical significance at *p* < 0.01.

The PNAs employed in this study were designed according to our standardized protocols, using the following criteria: (a) length of 18 bp, suitable for efficient synthesis also on large scale; (b) lack of self-complementarity both in antiparallel and parallel orientation; (c) minimal length of complementary sequences in mRNA, as evaluated by BLAST search; (d) when possible, targeting of the “seed region”, which is an essential element for miRNA function. A carrier octaargine R8 peptide was conjugated at the N-terminus of the PNA chain because this causes an efficiency in the delivery which approaches 100% (i.e., uptake in 100% of the target cell population), as published elsewhere [24,25,26,27,28]; this conjugation is easily realized during PNA solid-phase synthesis using the same reagents and solvents. A control PNA (R8-PNA-a335-MUT) was obtained by scrambling the position of four nucleobases, thus leaving the same base composition. Mutated sequences were also analyzed using Blast search to assess possible interferences. Figure 3A reports the miR-335-5p binding sequences recognizing the 3′-UTR NHERF1 miR-335-5p binding sites, as well as the sequences of PNA selected in the present study. R8-PNA-a335-5p displays a fully complementary sequence with respect to R8-PNA-a335-MUT harboring four changes suppressing the hybridization, as shown in previous studies [33].

When bronchial epithelial Calu-3 cells were cultured in the presence of R8-PNA-a335 and of the mutated sequence R8-PNA-a335-MUT, a clear-cut result was obtained and depicted in Figure 3B. First of all, treatment of Calu-3 cells with R8-PNA-a335 leads to a sharp inhibition of miR-335-5p hybridization signals; second, the mutant R8-PNA-a335-MUT displayed no inhibitory effects. In addition, the representative data shown in Figure 3C demonstrate that the effects are fairly specific, as the hybridization specific signals for other miRNAs expressed in Calu-3 cells (for instance, miR-101-3p) were unchanged following R8-PNA-a335 (the data concerning miR-101-3p are shown in Figure 3C, together with the effects of a PNA against miR-101-3p on miR-335-5p hybridization signals). Altogether, these experiments support the concept that the effects of R8-PNA-a335 on miR-335-5p are sequence-specific.

### 3.5. Effects of the R8-PNA-a335 on NHERF1 in Calu-3 Cells

**Figure 4:**
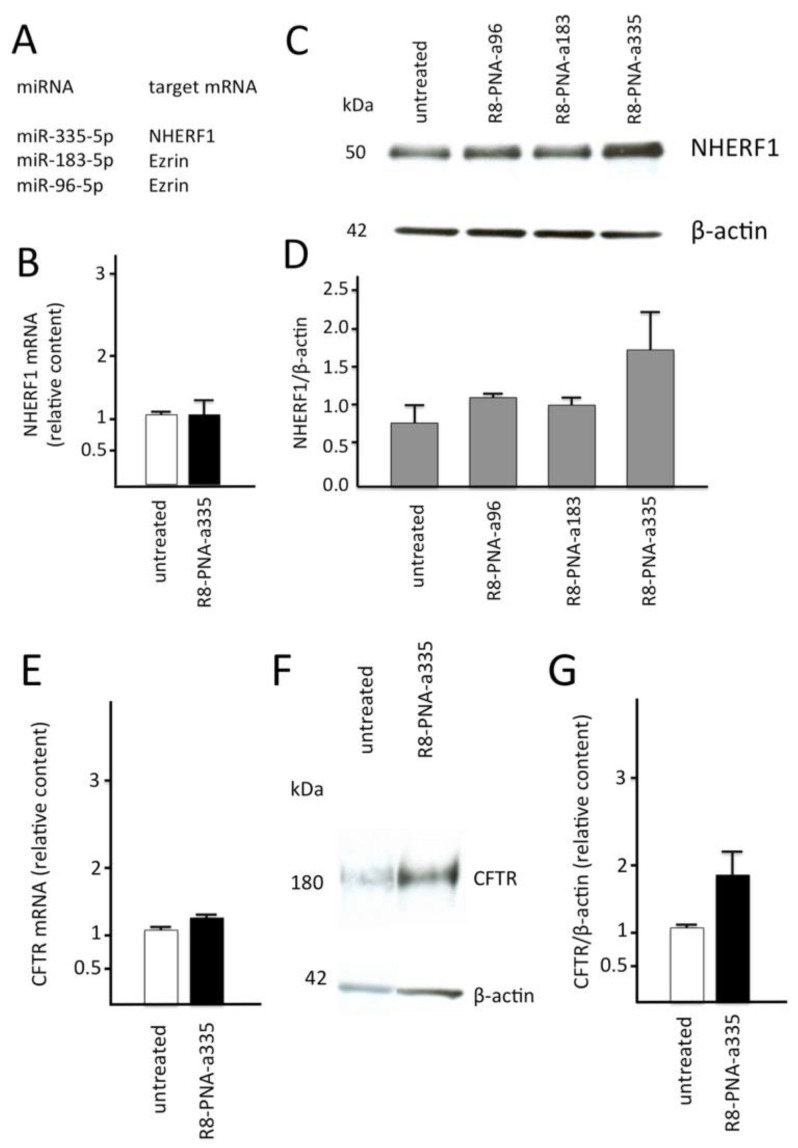
(**A**) The studied microRNAs and relative mRNA targets. Changes in *NHERF1* (**B**–**D**) and *CFTR* (**E**–**G**) gene expression analyzed by RT-qPCR (B,E) and Western blotting (**C**,**D**,**F**,**G**) in samples from Calu-3 cells cultured for 72 h in the absence or in the presence of R8-PNA-a335-5p, as indicated. The mRNA regulated by the targeted miRNAs are outlined in panel A. Results are expressed as mean ± standard error of the mean (SEM) of at least three independent experiments. Changes in the content of NHERF1 (**C**,**D**) and CFTR (**F**,**G**) relative to ß-actin (**C**,**D**,**F**,**G**) are indicated.

In Figure 4A, the mRNAs potentially targeted by miR-335-5p, miR-183-5p and miR-96-5p are indicated. MicroRNAs miR-183-5p and miR-96-5p display binding sites within the 3′-UTR sequence of *Ezrin* mRNA, while miR-335-5p binds to the 3′-UTR sequence of *NHERF1* mRNA. When Calu-3 cells were cultured in the presence of R8-PNA-a335, a clear effect was observed in *NHERF1* mRNA accumulation and NHERF1 production. In fact, the *NHERF1* mRNA content did not increase (see Figure 4B), while an increase in the NHERF1 protein was clearly detectable (Figure 4C,D) following Western blotting analysis. In all these experiments, the results were compared with those obtained using control untreated Calu-3 cells. These data are consistent with previous findings showing that miRNAs principally induce translational repression through 3′-UTR inhibition in mammals [59]. A recent model proposed that, in a sequential process, miRNA/mRNA interaction at the RISC inhibit translation at first and subsequently initiate mRNA degradation [60]. The sequential order of these two events might reflect kinetic differences between translational repression and mRNA decay rather than a cause-and-effect relationship between them [61,62,63]. The increase in NHERF1 protein expression depends on the PNA employed, since two PNAs against Erzin regulating miRNAs (R8-PNA-a183 and R8-PNA-a96, as outlined in Figure 4A) were not effective (Figure 4C).

### 3.6. Effects on CFTR mRNA and Protein 

When Calu-3 cells were cultured in the presence of the R8-PNA-a335, in addition to the NHERF1 up-regulation shown in Figure 4 (panels A–D), a clear effect was observed on CFTR production. Notably, only a low increase in *CFTR* mRNA was demonstrated after treatment of Calu-3 cells with the R8-PNA-a335 (Figure 4E). The hypothesis was that, despite the fact that miR-335-5p is predicted to interact with the 3′-UTR sequence of the *CFTR* mRNA [38], this interaction exhibits low efficiency in destabilizing *CFTR* mRNA. On the contrary, CFTR protein content was found to be sharply increased (Figure 4F,G). These experiments cannot tell whether increased CFTR content is due (a) to a translation effect of the PNA-a335 on the miR-335-5p mediated translational inhibition of CFTR or (b) to indirect effects possibly mediated by a PNA-a335 dependent NHERF1 up-regulation, or both. While additional PNA-a335 mediated effects cannot be ruled out and should be further studied, it has to be underlined that CFTR protein increase does not mean “per se” up-regulation of CFTR function. In order to have further information on these issues, functional studies have been considered.

### 3.7. Assessment of CFTR Functional Properties in the Presence of R8-PNA-a335

**Figure 5:**
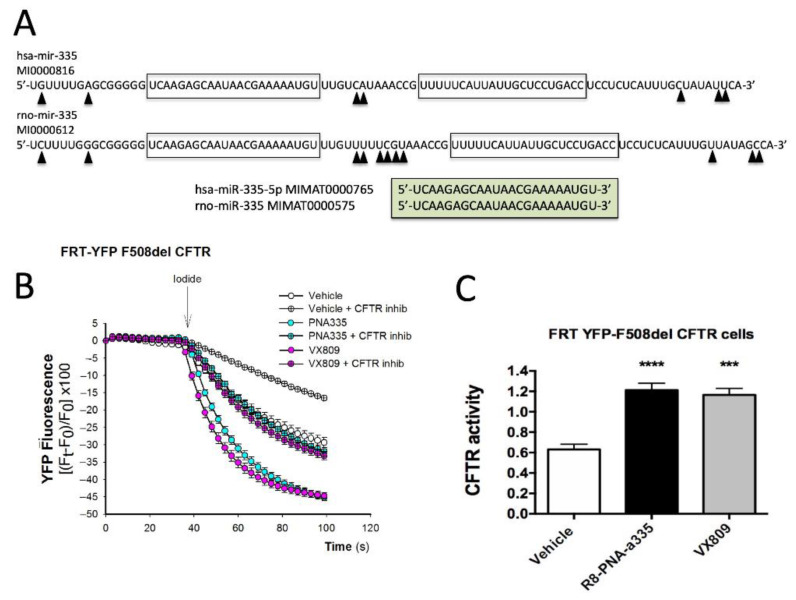
Effects of R8-PNA-a335 on CFTR function. (**A**) Both miR-335-5p and miR-335-3p (boxed sequences) are conserved when human (hsa) and rat (rno) pre-miR-335 sequences are compared. Arrowheads indicate mutated nucleotides. The fully homologous mature hsa and rno miR-335 sequences are shown in the light green box. B, C. Effects of R8-PNA-a335 on CFTR halide ion transport in FRT-YFP F508del CFTR cells. Cells were cultured untreated, or were treated with R8-PNA-a335 (4 μM) or VX 809 (5 μM) for 48 h. CFTR function was assessed by single-cell fluorescence imaging in the presence of the protein kinase A activator forskolin (20 µM) and genistein (50 µM). Representative traces are shown in panel (**B**). The summary of three independent experiments is shown in panel (**C**). Results are presented as transformed data to obtain the signal variation (Fx) relative to the time of addition of iodide, according to the equation: Fx ([Ft Fo]/Fo) 100, where Ft and Fo are the fluorescence values at the time t and at the time of addition of the iodide, respectively. Statistical comparisons were made using a nonparametric ANOVA test (*** *p* < 0.001, and **** *p* < 0.0001).

In order to evaluate whether R8-PNA-a335 rescues the F508del CFTR function, Cl- transport was studied in FRT cells co-expressing human F508del CFTR and yellow fluorescent protein (YFP H148Q/I152L). In this experimental cellular system, the high CFTR expression is dependent on a correction of the mutated F508del *CFTR* transcript encoded by the recombinant construct employed [40,41]. Firstly, we verified by RT-ddPCR that miR-335-5p is produced by FRT cells (Supplementary Figure S5) and that a 100% homology (and therefore ability to be targeted by the R8-PNA-a335) does exist between human and rat miR-335-5p (Figure 5A). Then, FRT cells were treated for 48 h with or without 4 μM R8-PNA-a335 or VX 809 (Lumacaftor) (5 μM) and CFTR-dependent Cl- transport measured by single cell digital imaging (Figure 5B,C). As shown in Figure 5B,C, we found that R8-PNA-a335 rescued CFTR-dependent Cl- transport. Interestingly, this effect was remarkable, since this miR-335 dependent rescue was obtained with an efficiency similar to that of VX 809. Since the PNA-a335 mediated rescue of the CFTR biological activity in this system cannot be simply caused by an increase in mutated CFTR, we hypothesize that this rescue of CFTR activity is associated with the up-regulation of NHERF1, which is known to be able to cause the rescue of the deltaF508CFTR functional expression [18,64,65].

### 3.8. Lack of Antiproliferative and Pro-Apoptotic Effects of R8-PNA-a335 on Calu-3 Cells

**Figure 6:**
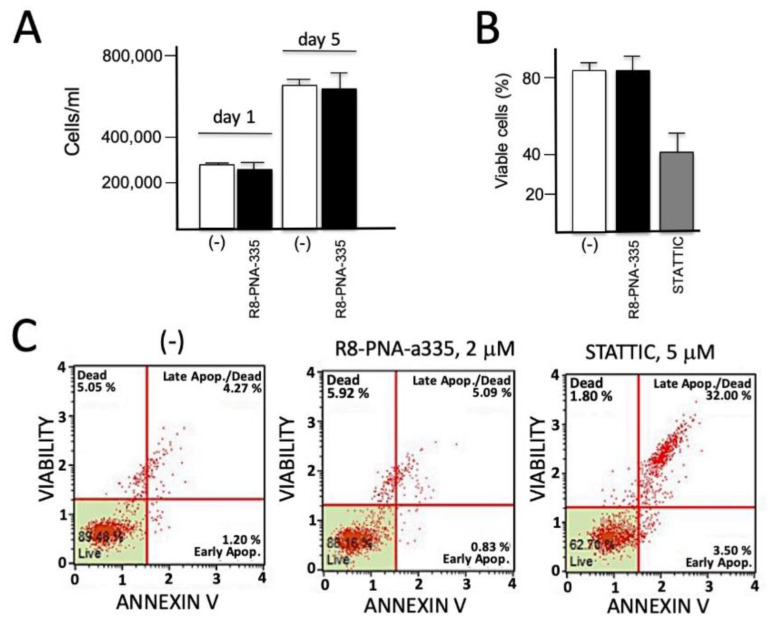
Effects of R8-PNA-a335 on cell growth, vitality, and apoptosis. (**A**) Calu-3 cells were cultured in the absence or in the presence of R8-PNA-a335 (4 µM) for different days and the cell number/mL determined. (**B**,**C**) Effects of R8-PNA-a335 on vitality (**B**) and apoptosis (**C**) of Calu-3 cells. Vitality was determined by trypan blue analysis of dead cells; apoptosis by the Annexin V & Dead Cell Kit (Merk Millipore). Red dots = cells. The green boxes include live, non apoptotic cells.

The results shown in Figure 6 were obtained in order to verify whether R8-PNA-a335 was to some extent cytotoxic. The results obtained demonstrate that the PNA tested did not exhibit antiproliferative effects (panel A), did not reduce the extent of viable cells (panel B), and did not induce apoptosis when compared to the proapoptotic compound Stattic (Selective STAT3 inhibitor, panel C). In these experiments, Calu-3 cells were cultured for 72 h in the absence, in the presence of R8-PNA-a335 or with the apoptotic inducers Stattic (5 μM) combined to 10% DMSO. After this treatment, the cell number/mL was determined, the dead cells were measured, and the possible induction of apoptosis was detected. The Annexin V and Dead Cell assays were performed with the Muse Cell Analyzer (Merck Millipore) method. The results obtained are shown in Figure 4C and demonstrate that apoptosis was not induced by PNAs targeting miR-335-5p, whereas under the same conditions, an increase in apoptosis was observed using Stattic with 10% DMSO.

### 3.9. R8-PNA-a335 Treated Calu-3 Cells: miRNome Profile Studied by Next-Generation Sequencing (NGS)

Despite the fact that the treatment of Calu-3 with the R8-PNA-a335 leads to a sharp inhibition of miR-335-5p (Figure 3) and a lower effect on other miRNAs (i.e., miR-101-3p, see Figure 3C), we were interested in verifying the effects of PNA treatment on the overall miRNome. To this end, RNA from Calu-3 cells, either untreated or treated with 4 μM R8-PNA-a335, was analyzed by NGS, using the Illumina NextSeq500 platform and NextSeq^®^ 500/550 High Output Kit v2 (see the Materials and Methods section). By means of the small RNA-Seq approach, thousands of small RNA and miRNA sequences can be analyzed with unprecedented sensitivity and dynamic range. In particular, differential expression of all small RNAs in any sample can be measured and changes in content can be characterized without prior sequence or secondary structure information.

**Figure 7:**
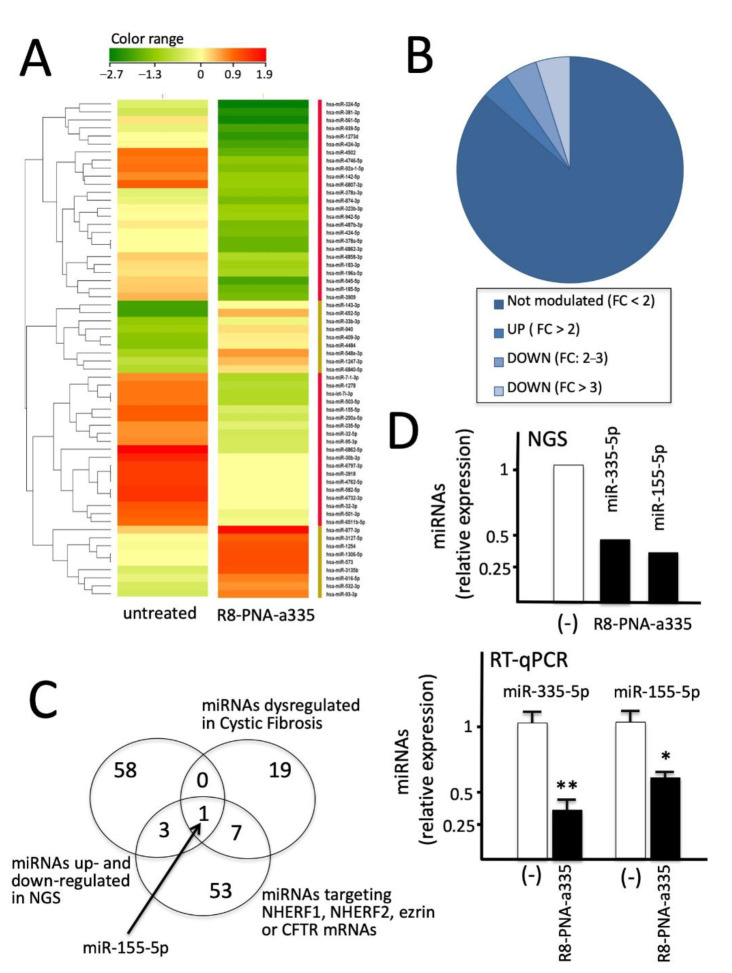
Effects of R8-PNA-a335 on miRNome profile analyzed by NGS sequencing. (**A**) Calu-3 cells were cultured in the absence or in the presence of R8-PNA-a335 for 48 h, RNA was extracted and NGS performed as described in the Materials and Methods section. (**B**) Summary of the number of miRNAs not modulated, or up- (FC > 2) and down- (FC: 2–3; FC > 3) modulated. (**C**) Venn diagram showing miR-155-5p as the only miRNA in common with the three lists (miRNAs up- and down-regulated in NGS, miRNAs dysregulated in CF and miRNA targeting *NHERF1, NHERF2, Ezrin* or *CFTR* mRNAs). (**D**) Validation of the R8-PNA-a335 mediated down-regulation of miR-155-5p by RT-qPCR. Data were derived by three independent experiments. Statistical comparisons were made using a nonparametric ANOVA test (* *p* < 0.05, ** *p* < 0.01).

Figure 7 shows some of the results obtained (the differentially expressed miRNAs are shown in panel A). By analyzing the NGS data imposing a two-fold change (FC) value most of the miRNA expressed (390/451, 86.5%) exhibited changes below this threshold value. On the contrary, 18 miRNAs (3.9%) were found to be up-regulated, and 43 miRNA were down-regulated (9.6%) (Figure 7B and Supplementary Table S1). Among the down-regulated miRNAs, 21 (4.7%) exhibited an FC between two and three, and 22 (4.9%) exhibited an FC greater than three. Fully in agreement with the data shown in Figure 3, miR-335-5p was found among the down-regulated miRNAs, demonstrating that two different experimental approaches (RT-qPCR and NGS) lead to similar results. When the NGS data (Supplementary Table S1) were compared with the list of miRNAs dysregulated in cystic fibrosis (Supplementary Table S2) and the list of miRNAs putatively able to target the 3′-UTR sequences of mRNA involved in CFTR expression (*NHERF1*, *NHERF2*, *Ezrin* and *CFTR* mRNAs) (Supplementary Table S3), only one miRNA was found to be present in all the lists, namely miR-155-5p. This is of interest since miR-155-5p deserves consideration for several reasons: (a) it can target NHERF2, another CFTR positive regulator [66]; (b) it has been demonstrated to be involved in cystic fibrosis by regulating FOXO1 [67]; (c) it is expected to regulate TCF4 [68], a transcription factor positively affecting CFTR expression [69]; and (d) promotes inflammatory responses following miR-155 dependent hyperactivation of IL-8 [70]. In consideration of this observation, an RT-qPCR analysis was therefore conducted, leading us to fully confirm the NGS results (Figure 7D). These data sustain the concept that a single PNA is able to down-regulate not only the specific miRNA (in our case miR-335-5p), but also other miRNAs (for instance miR-155-5p), which might be involved in the regulation of the same pathway(s) (in our case, CFTR expression) or other pathways important in CF (i.e., the hyperinflammatory state characterized by overexpression of pro-inflammatory genes, including *IL-8*). In conclusion, the down-regulation of miR-155 was confirmed using three independent approaches, i.e., NGS (Figure 7, panels A and D), RT-qPCR (Figure 7, panel D), and digital RT-ddPCR (Supplementary Figure S6).

## 4. Discussion

Since the demonstration that microRNAs are deeply involved in the regulation of CFTR and scaffolding proteins (such as NHERF1, NHERF2 and Ezrin), great attention has been dedicated to possible CFTR alteration of gene expression by targeting miRNAs causing down-regulation of CFTR and associated proteins. For instance, PNA-mediated inhibition of miR-145-5p (which down-regulates CFTR) leads to an increase in CFTR in the Calu-3 model system [36].

The results here described show that the CFTR scaffolding protein NHERF1 can be up-regulated by a PNA-mediated inhibition of miR-335-5p (Figure 4C), predicted to bind to the *NHERF1* mRNA 3′-UTR sequence. At the same time, up-regulation of CFTR was observed (Figure 4F,G). We therefore propose miR-335-5p targeting as a strategy to increase CFTR. As miRNA binding sites have been also predicted in the 3′-UTR of *CFTR* mRNA [38], we cannot exclude the possibility that miR-335-5p could act also by direct binding to *CFTR* mRNA. In any case, the CFTR increase through a miR-335-5p dependent up-regulation of NHERF1 is confirmed by the experiments depicted in Figure 5 and carried out using the rat FRT-YFP F508del CFTR cell line, producing a mutated F508del *CFTR* mRNA needing to be suitably corrected to allow CFTR function. 

**Figure 8:**
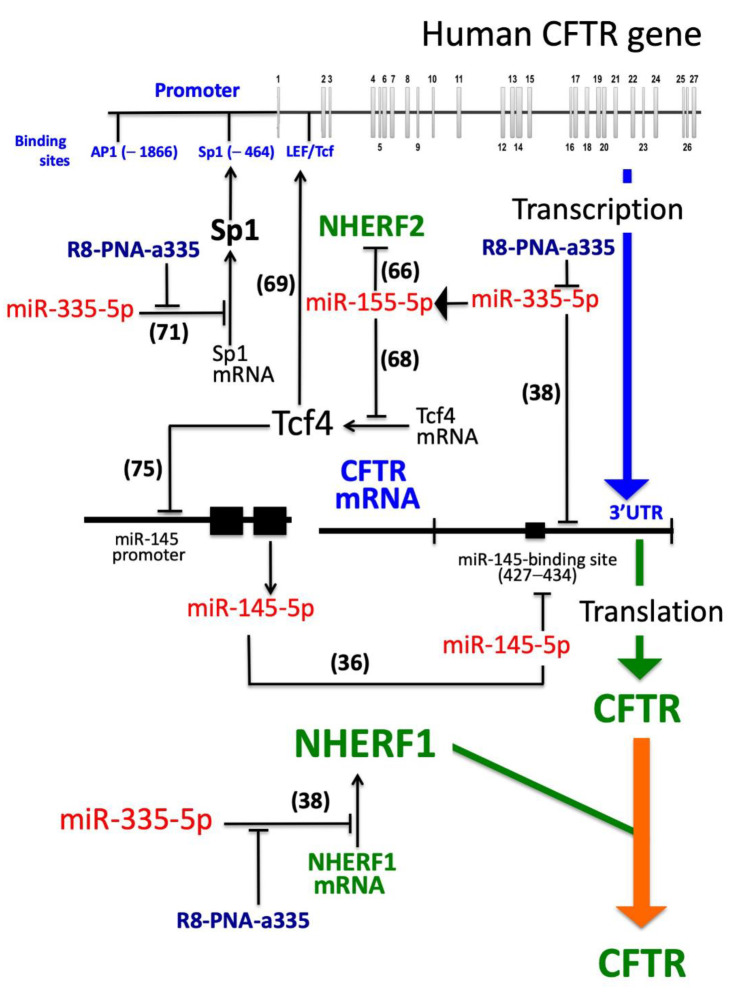
Proposed model for the interplay between transcription factors, NHERF1, CFTR regulation and miR-335-5p regulated molecules. The published information concerning specific steps are indicated in parenthesis. The effects of R8-PNA-a335 on miR-335-5p are presented in Figure 3. The effects of R8-PNA-a335 on miR-155-5p are described in Figure 7. The effects of R8-PNA-a335 on NHERF1 and CFTR are presented in Figure 4. MicroRNAs and PNAs are in red- and blue-marked, respectively. The colored arrows represent transcription (blue), translation (green) and processing (orange).

While the efficiency of this strategy on CF caused by stop-codon mutation of the *CFTR* gene should be verified, this approach is expected to give appreciable results in CF cells carrying mutations impairing the processing or stability of the CFTR protein, supporting an interesting strategy for personalized therapy in precision medicine. We cannot exclude, however, the multiple effects of PNA-mediated inhibition of miR-335-5p involving other mRNA targets; for instance, miR-335-5p negatively regulates the mRNA for Specific protein 1 (Sp1), a transcriptional factor up-regulating CFTR transcription [71,72]. Therefore, PNA-mediated inhibition of miR-335 might lead to increased Sp1 and up-regulated *CFTR* transcription (see the proposed mode of action depicted in Figure 8). Despite the fact that our study does not clarify this specific point, future experiments based on miR-335-5p transfections might help in identifying which of these possible pleiotropic effects of PNA-a335 are occurring. 

With respect to the clinical relevance of miRNA targeting, it is expected that decreased availability of miRNAs (anti-miR approach) is associated with an accumulation of target mRNAs; conversely, increased expression of miRNAs (miRNA mimicking) is expected to decrease the expression of the target mRNAs. In this context, peptide nucleic acids (PNAs) represent molecules with strong antimiRNA activity. As far as clinical trials based on miRNA therapeutics are concerned (www.clinicaltrials.gov), examples include a Phase 2 (miravirsen by Santaris Pharma, NCT01200420) and one Phase I (RG-101 by Regulus Therapeutics, NCT00980161) trial, both based on targeting miR-122 for therapy of Hepatitis C virus infection.

While the results here presented are a proof-of-principle that PNA-based targeting miRNAs regulating CFTR scaffolding proteins (such as NHERF1) might lead to CFTR up-regulation, several considerations should be discussed.

First of all, the effects of PNA-a335 on NHERF1 do not lead to up-regulation of the target *NHERF1* mRNA. This is not unexpected, since the same effects has been found by miRNA-dependent targeting of other mRNAs [60,61,62,63]. In the case of antagomiRNAs against miR-335-5p, Yao et al. [73] found that a miR-335-5p inhibitor increased the protein expression levels of SGK3 in KGN cells, exhibiting lower effects on *SGK3* mRNA. In agreement, Zhang et al. [74] found that miR-335-5p targeting did not have any effect on *DKK1* mRNA levels, whereas it increased DKK1 protein levels. A possible explanation for the hypothesis on the effect of PNA-a335 on *NHERF1* mRNA is the lack of full complementarity between miR-335-5p and the relative 3′UTR region of the *NHERF1* mRNA (see Figure 2A). 

Second, since several miRNAs can bind to the *NHERF1* mRNA, targeting other miRNAs might lead to even more efficient up-regulation of NHERF1 and CFTR. Third, possible combined effects of PNAs targeting different miRNAs involved in NHERF1 regulation should be considered in the future in order to obtain the highest level of CFTR induction. Fourth, a combined PNA-based approach for targeting miRNAs regulating NHERF1 and CFTR should be also considered. Finally, our observations should be confirmed in other CF cellular model systems.

As far as these issues are concerned, another important conclusion derived from the results here presented is that the miRNome is to some extent affected by the treatment of target cells with anti-miRNA PNAs. This is demonstrated by the results depicted in Figure 6, showing that some miRNAs are up-regulated and some miRNAs are down-regulated following treatment of Calu-3 cells with the R8-PNA-a335. While the modulated miRNAs are only a large minority of the overall Calu-3 expressed miRNAs detectable by the NGS approach (13.6%), this finding further sustains the concept that combined treatments of target cells with sub-optimal concentrations of synergistic antagomiRNAs should be taken in great consideration, being one of the strategies to limit off-target effects. On the other hand, the data obtained support the concept that specific miRNA inhibition (in our case inhibition of miR-335-5p) might be accompanied by co-inhibition of other miRNAs. This might be of great interest in using one PNA molecule affecting different miRNAs, one directly (miR-335-5p) and the other one indirectly (miR-155-5p, see Figure 7). While the mechanism of action of miR-335-5p on miR-155-5p expression remains to be elucidated, we would like to comment that miR-miR-155-5p is responsible for hyperactivating IL-8, thereby promoting CF inflammation. Therefore, PNA-mediated targeting of miR-335-5p in Calu-3 cells is expected to lead, in addition to the up-regulation of NHERF1 and CFTR (Figure 4), to the down-regulation of IL-8, possibly combining two activities of great interest in CF therapy, i.e., CFTR potentiation and anti-inflammatory effects. Considering the effect of the R8-PNA-a335 on miR-155-5p, two additional mechanisms can be hypothesized (summarized in Figure 8): (a) an up-regulation of NHERF2, mediated by the decrease in miR-155-5p and (b) an up-regulation of TCF4, which, in addition to CFTR up-regulation, is a potent repressor of another CFTR-regulating miRNA, miR-145-5p [75]. In this specific case, down-regulation of miR-145 might lead to increased expression of CFTR, as recently published by our research group, using a PNA targeting miR-145-5p [36].

In conclusion, the finding that PNA-mediated down-regulation of a specific microRNA might be associated with alteration of a sub-set of other miRNAs should be considered during the development of therapeutic protocols, since it might have an impact on the biological outcomes.

A further issue (which is shared by several other anti-miRNA approaches) is the delivery of PNAs to the tissue to be corrected, i.e., the lung of CF patients. In this respect, some very interesting approaches are available. For instance, composite nanoparticles (NPs) of the poly (lactic-co-glycolic acid) (PLGA) and cationic polymer polyethyleneimine (PEI) [76] are efficient alternatives to viral and liposomal vectors for the pulmonary delivery of DNA.

Finally, an interesting possibility for the PNA-dependent modulation of miRNA involved in the regulation of CFTR expression is to combine their activity with that of drugs already employed for personalized therapy of CF [43,77,78,79,80,81,82,83], such as, for instance, the CFTR correctors VX809 and TMA [43,75,76,77]. This combined treatment might lead to unmet levels of functional CFTR production in CF cells, through the combination of two complementary activities, i.e., the miR-335-5p mediated up-regulation of CFTR production and the VX809 (or TMA)-dependent correction.

## Data Availability

The data presented in this study are available on request from the corresponding author.

## Supplementary Materials

The following are available online at https://www.mdpi.com/2227-9059/9/2/117/s1, Figure S1: UPLC-ESI/MS analysis of R8-PNA-a335, Figure S2: UPLC-ESI/MS analysis of R8-PNA-a335-MUT, Figure S3: UPLC-ESI/MS analysis of R8-PNA-a96, Figure S4: UPLC-ESI/MS analysis of R8-PNA-a183, Figure S5: Expression of miR-335-5p and miR-155-5p in FRT-F508del cells, Figure S6: Effects of R8-PNA-a335 treatment of Calu-3 cells on miR-155 expression, Table S1: R8-PNA-a335 treated Calu-3 cells: up-regulated and down-regulated miRNAs (fold change threshold: 2), Table S2: List of microRNAs dysregulated in Cystic Fibrosis, Table S3: MicroRNAs targeting *CFTR*, *NHERF1*, *NHERF2* and *Ezrin* mRNAs.
