# Supplementary material for: A Peptide-Nucleic Acid Targeting miR-335-5p Enhances Expression of Cystic Fibrosis Transmembrane Conductance Regulator (CFTR) Gene with the Possible Involvement of the CFTR Scaffolding Protein NHERF1
Source: Biomedicines. 2021 Jan 26;9(2):117. doi: 10.3390/biomedicines9020117 (PMC7911309; doi:10.3390/biomedicines9020117)
Supplement: Supplementary file 1 [file biomedicines-09-00117-s001.pdf]

biomedicines

Increased expression of the Cystic Fibrosis Transmembrane Conductance Regulator (*CFTR*) gene by a peptide-nucleic acid (PNA) targeting the NHERF1 regulator microRNA miR-335-5p

Anna Tamanini [1,], Enrica Fabbri [2,**], Tiziana Jakova [3], Jessica Gasparello [2], Alex Manicardi [3,#], Roberto Corradini [3], Alessia Finotti [2,4], Monica Borgatti [2,4], Ilaria Lampronti [2,4], Silvia Munari [1], Maria Cristina Dechecchi [1], Giulio Cabrini [4,5] and Roberto Gambari [2,4*]**

1 Section of Molecular Pathology, Department of Pathology and Diagnostics, University-Hospital of Verona, Italy;
2 Department of Life Sciences and Biotechnology, University of Ferrara, Italy;
3 Department of Chemistry, Life Sciences and Environmental Sustainability, University of Parma, Italy;
4 Research Center on Innovative Therapies for Cystic Fibrosis, University of Ferrara, Italy;
5 Department of Neurosciences, Biomedicine and Movement, University of Verona, Italy.

* Correspondence: gam@unife.it; Tel.: +39-0532-974443 (R.G.)
** Co-first author
\# current address: Department of Organic and Macromolecular Chemistry, Ghent University, Krijgslaan 281 S4, B-9000 Gent (Belgium)

Keywords: Peptide nucleic acids; cystic fibrosis; microRNAs; miR-335-5p; miRNA targeting; delivery; NHERF1; CFTR.

SUPPLEMENTARY MATERIAL

1. Supplementary methods

1.1. ddPCR assays

300 ng of total RNA were reverse transcribed using TaqMan miRNA Reverse Transcription Kit (Applied Biosystems, Thermo Fischer Scientific, Waltham, MA, USA) according to manufacturer's manual. The obtained cDNA was used for ddPCR (droplet digital PCR) analysis. 1.3 µL of cDNA, undiluted (for miRNAs quantification in FTR-F508del) or diluted 1:10 (for Calu-3 cells treated with R8-PNA-a335) were amplified in a final volume of 20 µL, containing ddPCR Supermix for Probes (no dUTP) 2X (Bio-Rad) and TaqMan miRNA 20X (Applied Biosystem, Thermo Fischer Scientific). The amplification mixture was loaded in 96-well PCR plate (Eppendorf, Amburgo, Germany) and the emulsion was automatically obtained using the Automated Droplet Generator (AutoDG, Bio-Rad). 40 µL of droplets were generated and automatically transferred to new 96-well PCR plate, that was heat-sealed using PX1 PCR Plate Sealer (Bio-Rad) and amplified in thermal cycler. The following thermal cycler conditions were used: 95°C for 10 min, 40 cycles of 95°C for 15 seconds and 60°C for 1 minute (ramp rate 2.5°C/sec) and a final step of 98°C for 10 minutes to inactivate the enzyme. Droplets were analyzed using QX200 Droplet Reader (Bio-Rad), and data analysis was performed with QuantaSoft version 1.7.4 (Bio-Rad) [1,2].

Table 1: employed probes

Assay name	Assay ID
Hsa-miR-335-5p	000546
Hsa-miR-155-5p	002622

2. Supplementary Figures

2.1. Supplementary Figures related to PNA characterization

Supplementary Figure S1.

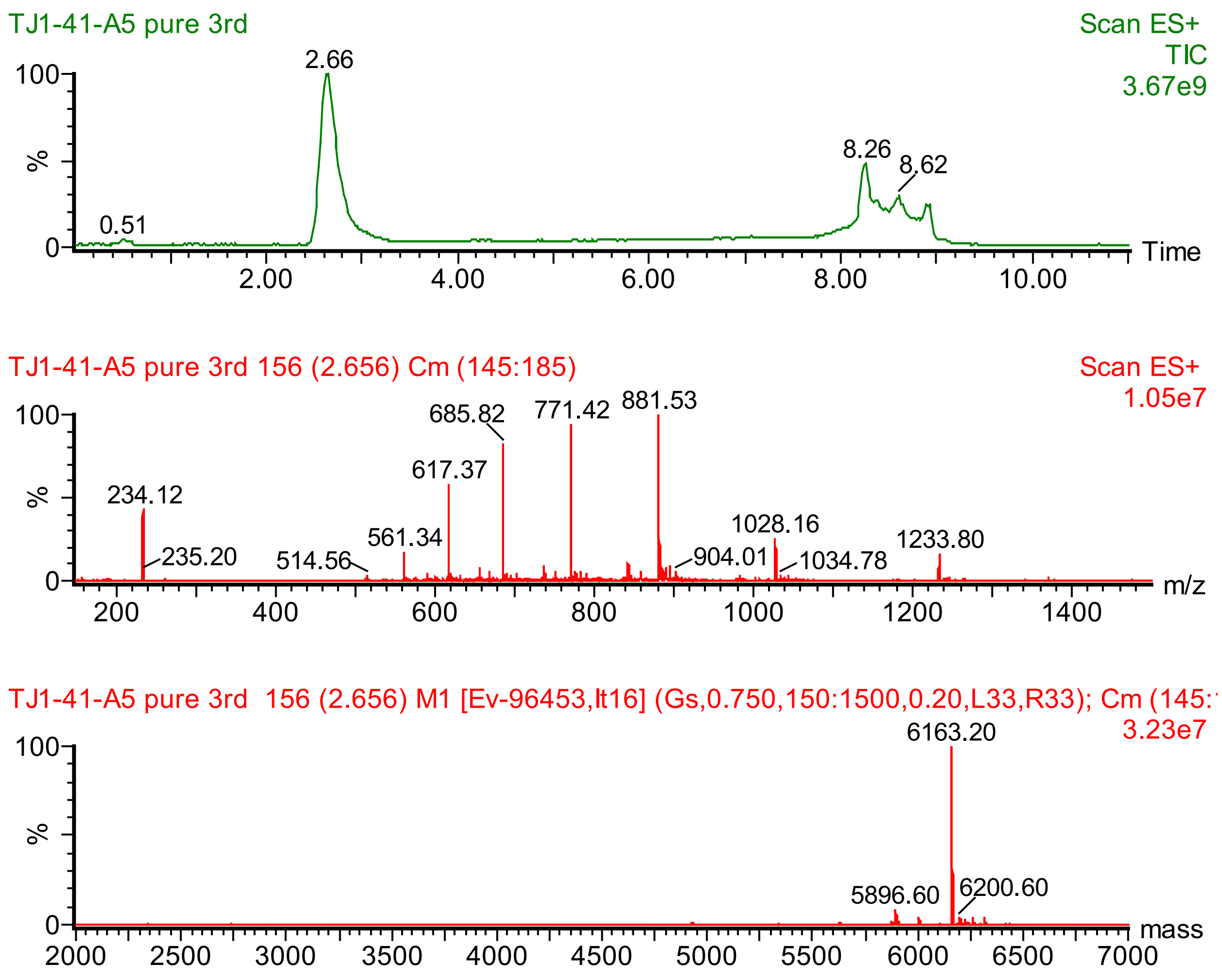

UPLC-ESI/MS analysis of R8-PNA-a335. Above UPLC chromatogram, middle: ESI-MS spectrum of peak at 2.66 min; below: mathematical deconvolution of the multicharged signals. Conditions are as indicated in the Materials and Methods section [3].

Supplementary Figure S2.

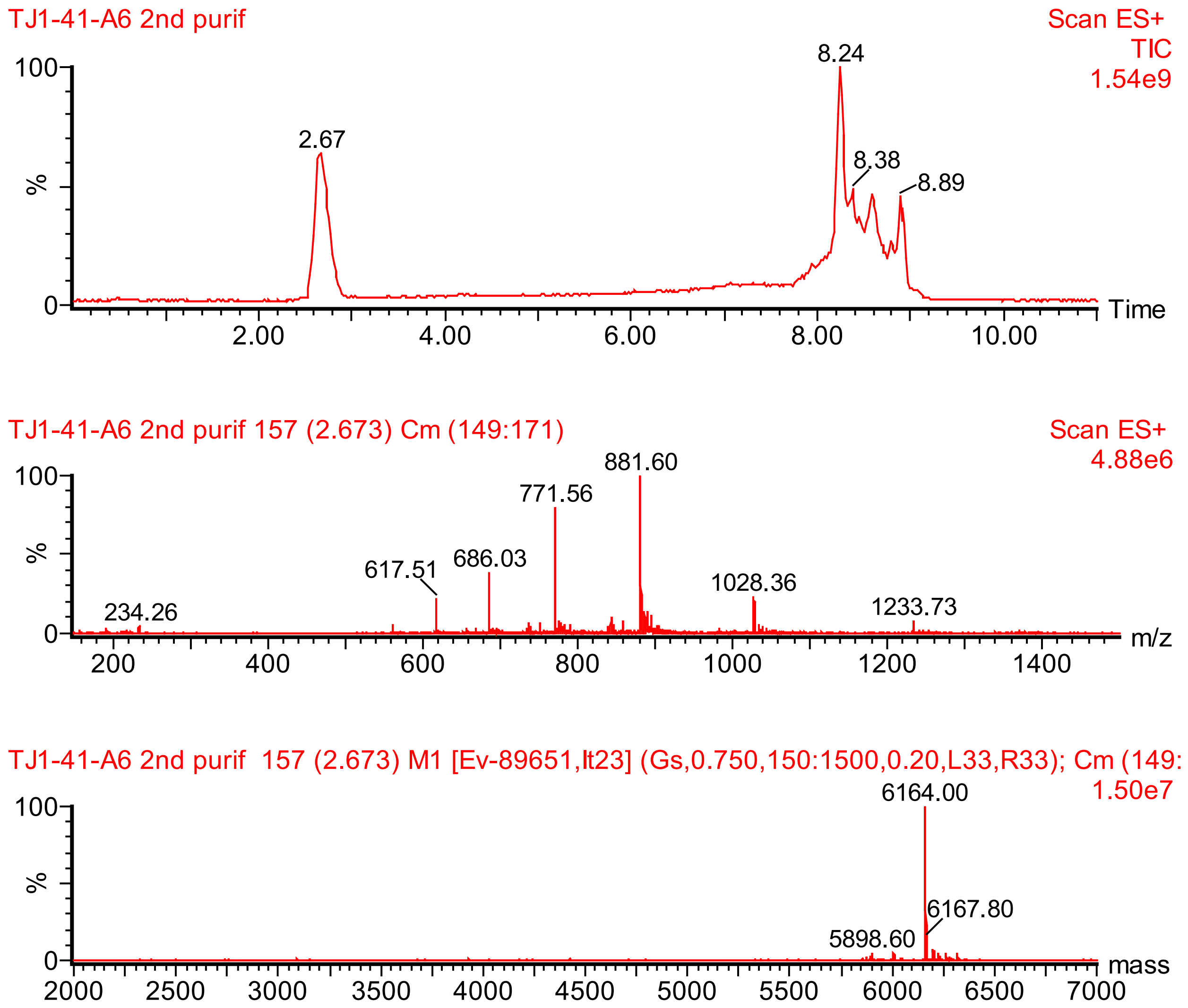

UPLC-ESI/MS analysis of R8-PNA-a335-MUT. Above UPLC chromatogram, middle: ESI-MS spectrum of peak at 2.67 min; below: mathematical deconvolution of the multicharged signals. Conditions are as indicated in the Materials and Methods section [3].

Supplementary Figure S3.

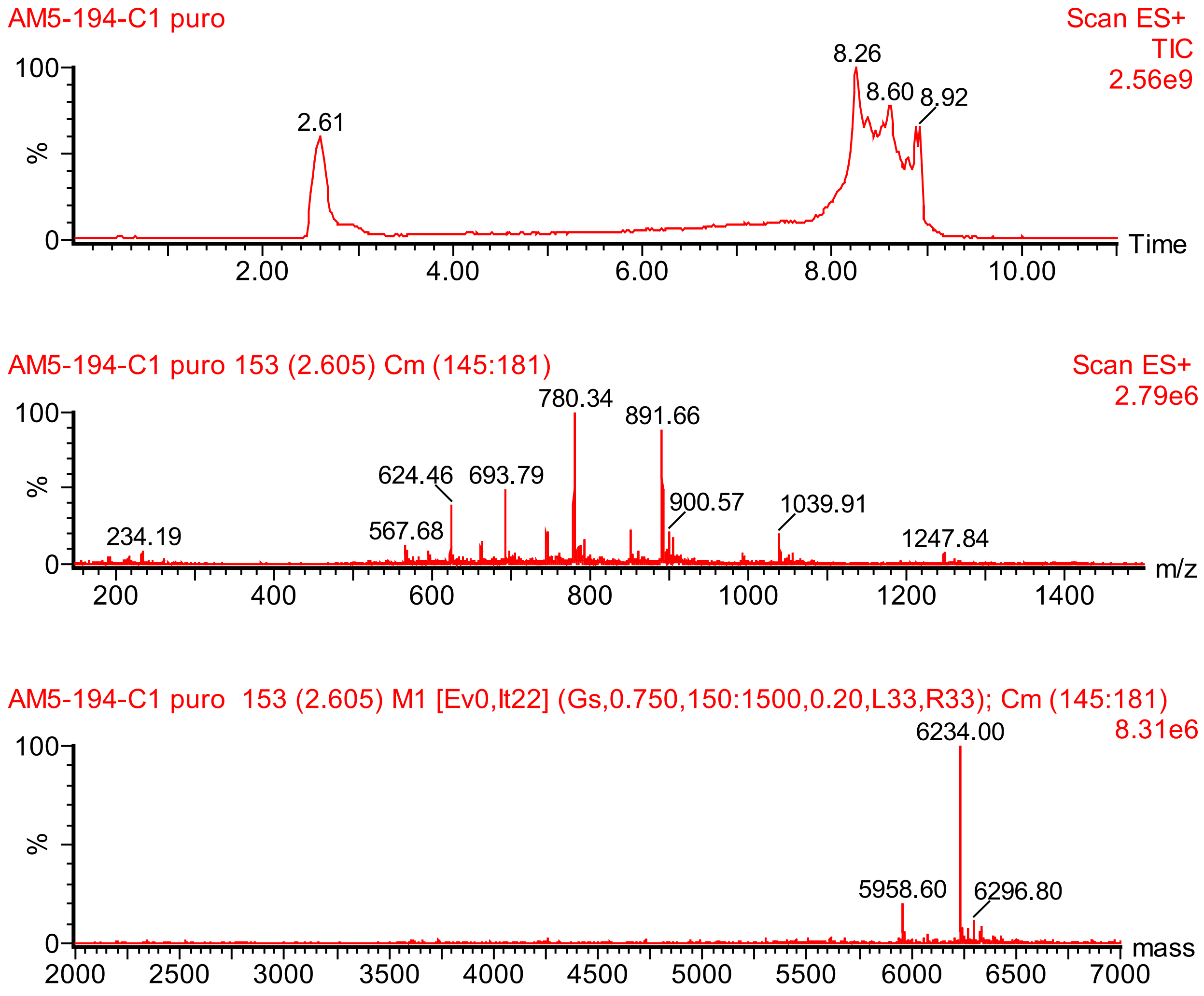

UPLC-ESI/MS analysis of R8-PNA-a96. Above UPLC chromatogram, middle: ESI-MS spectrum of peak at 2.61 min; below: mathematical deconvolution of the multicharged signals. Conditions are as indicated in the Materials and Methods section [3].

Supplementary Figure S4.

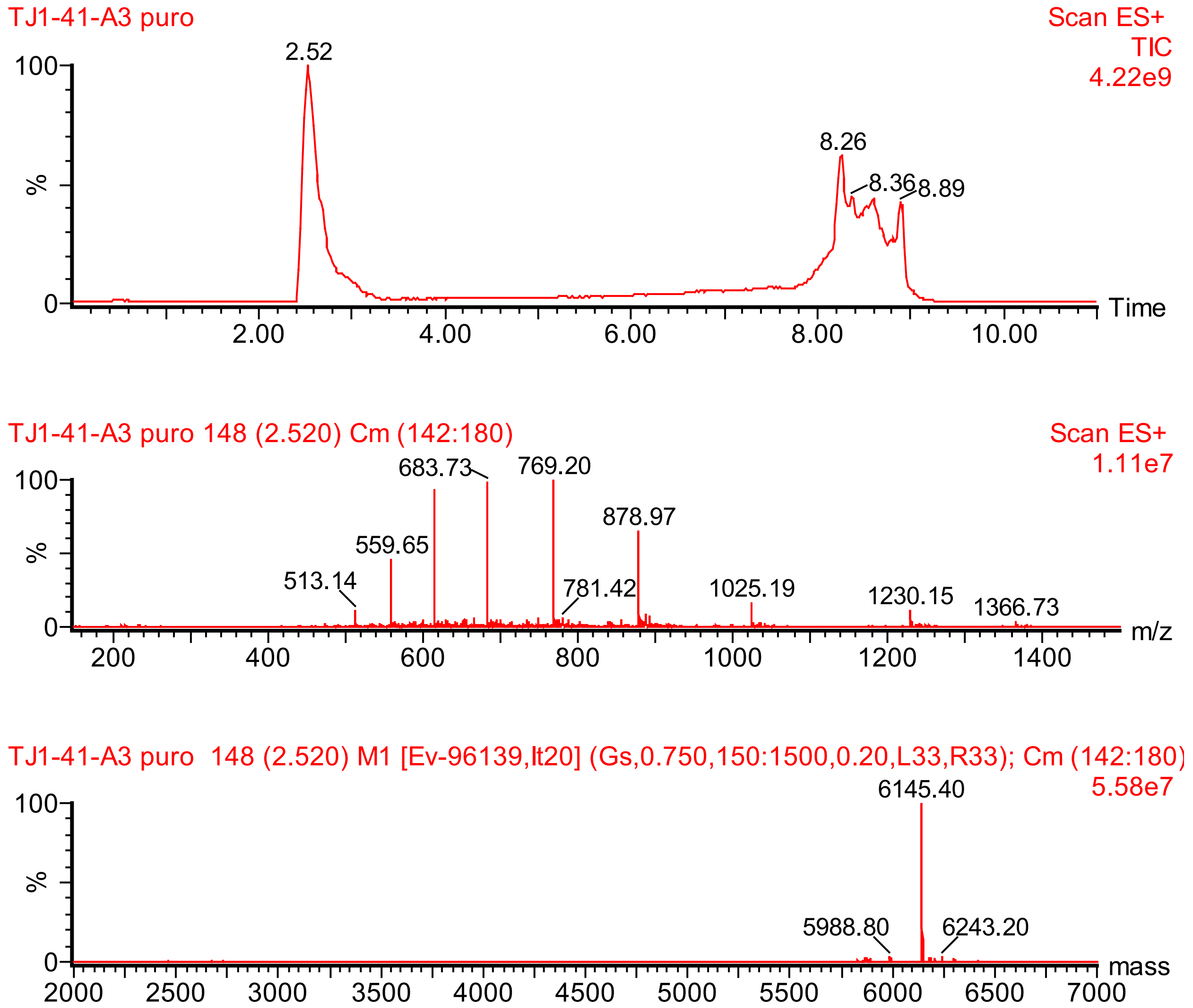

UPLC-ESI/MS analysis of R8-PNA-a183. Above UPLC chromatogram, middle: ESI-MS spectrum of peak at 2.52 min; below: mathematical deconvolution of the multicharged signals. Conditions are as indicated in the Materials and Methods section [3].

2.2. Supplementary Figures related to RT-ddPCR.

Supplementary Figure S5.

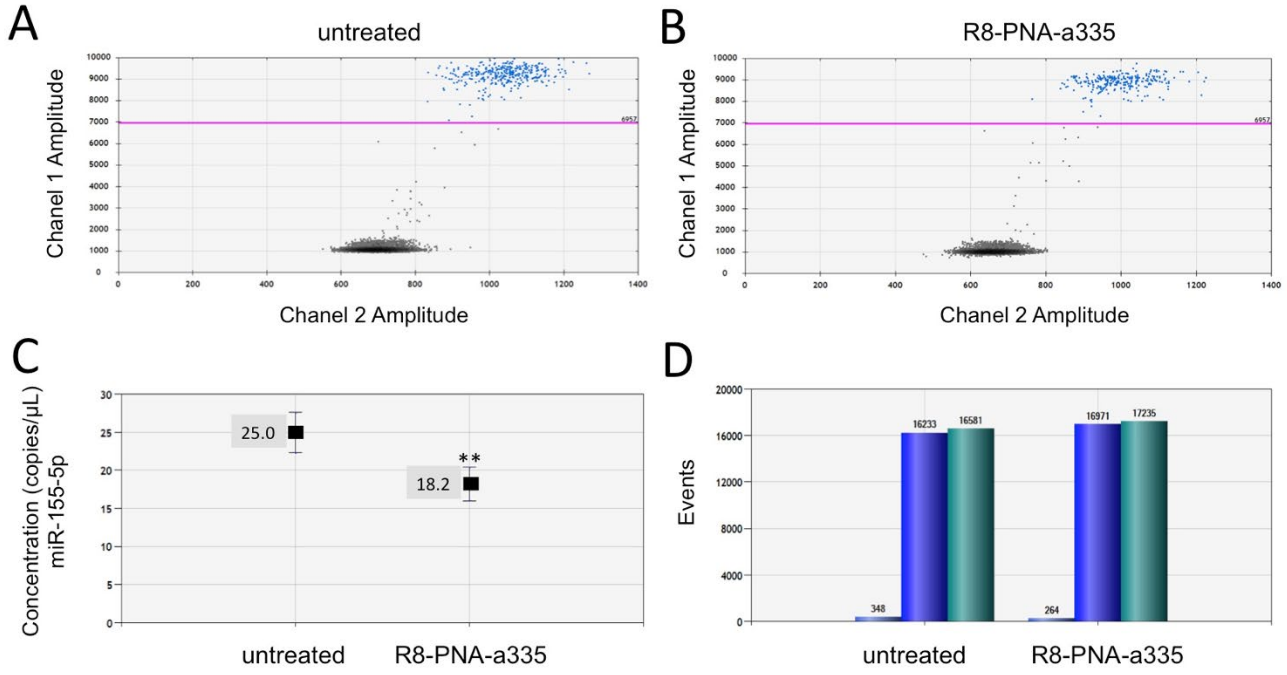

Effects of R8-PNA-a335 treatment of Calu-3 cells on miR-155 expression. The expression of miR-155 was verified using ddPCR, in both untreated cells and in cells treated with R8-PNA-a335 for 48 hours. In panels A and B plots are reported representative examples of obtained positive events for an untreated sample (left) or a sample derived from Calu-3 cells treated with R8-PNA-a335 (right). Blue dots indicate positive events, while negative events are represented by black spots. Analyzed cDNA has been diluted 1:10 before the analysis. C. Concentration plot: miR-155 concentration expressed as copies/µL was reported for each sample (untreated and treated with PNA-a335). D. Events plot: total analyzed events are reported in green box, negative events are indicated in blue box, while, the smaller light blue box indicates positive events. ** = $p < 0.01$ (n=3).

Supplementary Figure S6.

A
B
C
D

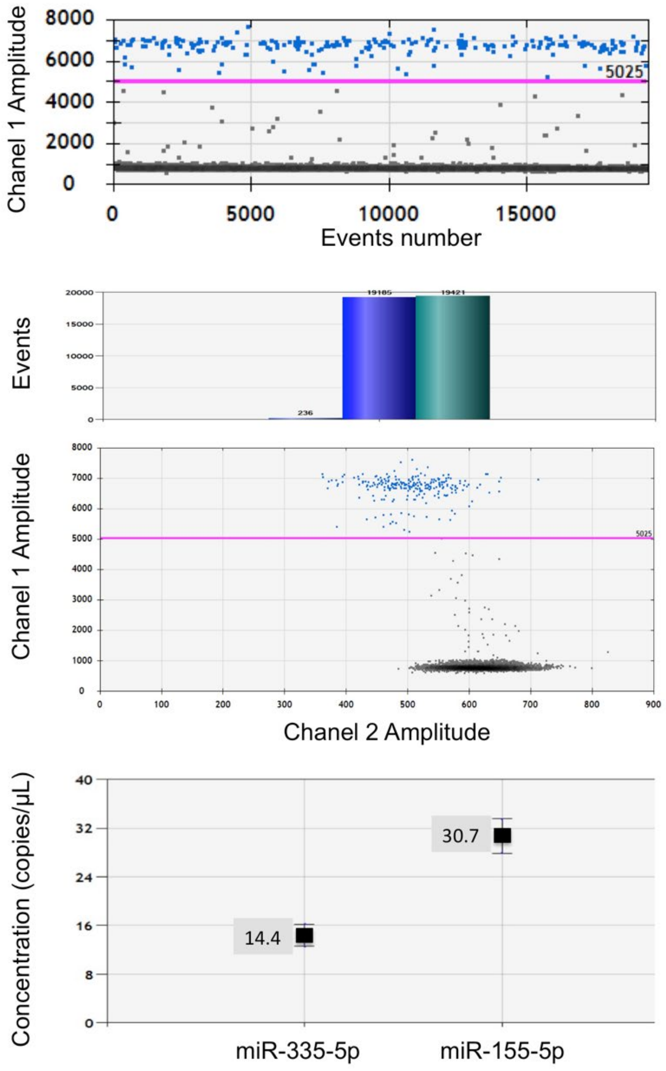

Expression of miR-335-5p and miR-155-5p in FRT-F508del cells. A. Representative 1D plot using miR-335-5p primers: blue spots indicate positive events. B. Event plot (miR-335-5p primers): light blue box positive droplets, blue box negative droplets and green box total droplets (positive plus negative droplets). C. Representative 2D plots (miR-335-5p primers). D. Concentration of miRNAs miR-335-5p and miR-155-5p in the FRT-F508del cell line; concentration is expressed as copies of miRNAs/µL of cDNA. The same amount of cDNA (1.3 µL) was employed.

Supplementary Table S1. R8-PNA-a335 treated Calu-3 cells: up-regulated and down-regulated miRNAs (fold change threshold: 2)

microRNAs	Fold Change	Regulation	microRNAs	Fold Change	Regulation
let-7i-3p	3,5557377	down	miR-501-3p	2,370492	down
miR-1247-3p	2,3623788	up	miR-503-5p	3,5557377	down
miR-1254	2,3623793	up	miR-532-3p	2,2498844	up
miR-1273d	4,7409835	down	miR-545-5p	5,0343513	down
miR-1278	3,3562343	down	miR-548e-3p	3,3748267	up
miR-1306-5p	2,2498846	up	miR-561-5p	6,518852	down
miR-142-5p	4,1483603	down	miR-573	2,2498844	up
miR-143-3p	4,218534	up	miR-582-5p	2,6668038	down
miR-155-5p	2,8972678	down	miR-616-5p	2,1092668	up
miR-183-3p	2,6668038	down	miR-6511b-5p	2,0741808	down
miR-185-5p	4,148361	down	miR-652-5p	5,9059477	up
miR-196a-5p	2,4297543	down	miR-6732-3p	2,666804	down
miR-200a-5p	3,160656	down	miR-6797-3p	2,5171754	down
miR-30b-3p	2,963115	down	miR-6807-3p	5,0343513	down
miR-3127-5p	2,024896	up	miR-6840-5p	2,2498846	up
miR-3135b	3,0373442	up	miR-6858-3p	2,5171754	down
miR-32-3p	2,1334429	down	miR-6862-3p	3,3562343	down
miR-32-5p	2,370492	down	miR-6862-5p	5,333607	down
miR-323b-3p	2,5171754	down	miR-7-1-3p	3,3562343	down
miR-324-5p	4,7409835	down	miR-874-3p	2,5171754	down
miR-335-5p	2,0567503	down	miR-877-3p	2,812356	up
miR-33b-3p	2,2498846	up	miR-92a-1-5p	5,333607	down
miR-378a-3p	2,0741808	down	miR-93-3p	2,53112	up
miR-378a-5p	3,3562343	down	miR-939-5p	3,3562343	down
miR-381-3p	3,3562343	down	miR-940	2,812356	up
miR-3909	4,195293	down	miR-942-5p	2,3704917	down
miR-3918	2,5171754	down	miR-95-3p	2,370492	down
miR-409-3p	2,9529736	up			
miR-424-3p	4,148361	down			
miR-424-5p	2,963115	down			
miR-4484	2,9529736	up			
miR-4502	6,518852	down			
miR-4746-5p	4,7409835	down			
miR-4762-5p	2,5171754	down			
miR-487b-3p	3,3562343	down			

Supplementary Table S2. List of microRNAs dysregulated in Cystic Fibrosis

microRNA	Regulation	Experimental model system
Let-7e-5p	Up	DeltaF508-CFTR expressing cells
miR-101-3p	Up	bronchial brushing (CF vs non-CF)
miR-125b-5p	Up	DeltaF508-CFTR expressing cells
miR-125b-5p	Up	DeltaF508-CFTR expressing cells
miR-126-3p	Down	bronchial brushing (CF vs non-CF)
		airway epithelial cultures (CF vs non-CF)
miR-1343	Down	16HBE14o-
miR-138-5p	Up	airway epithelial cultures (CF vs non-CF)
miR-144-3p	Down	16HBE14o- cells
miR-145-5p	Up	bronchial brushing (CF vs non-CF)
		CFBE41o- vs 16HBE14o-
		nasal cavity brushing (CF vs non-CF)
miR-146a-5p	Down	bronchial brushing (CF vs non-CF)
		CFBE41o- vs 16HBE14o-
miR-155-5p	Up	CFBE41o- vs 16HBE14o-
		IB3-1 CF vs IB3/S9
miR-16-5p	Down	IB3-1, F508del-CFTR HBE cells
miR-17-5p	Up	airway epithelial cultures (CF vs non-CF)
miR-181b-5p	Up	CFBE41o- vs 16HBE14o-
miR-221-3p	Up	CFBE41o- versus 16HBE14o-
miR-223-3p	Up	bronchial brushing (CF vs non-CF)
		CFBE41o- vs 16HBE14o-
miR-224-5p	Up	oral buccal mucosal epithelial cells
miR-31-5p	Down	CFBE41o- vs 16HBE14o-
miR-370-3p	Up	CFBE41o- vs 16HBE14o-
miR-384	Down	CFBE41o- and 16HBE14o-
miR-494-3p	Up	bronchial brushing (CF vs non-CF)
		CFBE41o- vs 16HBE14o-
		bronchial brushing (CF vs non-CF)
miR-509-3p	Up	airway epithelial cultures (CF vs non-CF)
miR-708-5p	Up	CFBE41o- vs 16HBE14o-
miR-93-5p	Down	IB3-1 cells (infected with Ps. Aeruginosa versus not infected)
miR-9-5p	Down	CFBE41o- vs 16HBE14o-
miR-99b-5p	Up	DeltaF508-CFTR expressing cells

From: Fabbri et al. [4].

Supplementary Table S3. MicroRNAs targeting *CFTR*, *NHERF1*, *NHERF2* and *Ezrin* mRNAs

**miRNA**	**Target**	**miRNA**	**Target**	**miRNA**	**Target**
let-7b-5p	NHERF1	miR-301b-5p	NHERF2	miR-5698	NHERF2
miR-101-3p	CFTR	miR-30a-5p	NHERF2	miR-593-3p	NHERF2
miR-1180-3p	Ezrin	miR-3120-5p	NHERF2	miR-600	CFTR
miR-1257	Ezrin	miR-328-3p	Ezrin	miR-607	CFTR
miR-125b-5p	NHERF2	miR-335-5p	CFTR, NHERF1	miR-615-3p	Ezrin
miR-132-3p	CFTR	miR-345-5p	NHERF2	miR-6719-3p	Ezrin
miR-1343-5p	NHERF2	miR-3622b-5p	Ezrin	miR-6730-3p	Ezrin
miR-144-3p	CFTR	miR-3660	Ezrin	miR-6812-3p	NHERF2
miR-145-5p	CFTR	miR-3677-5p	Ezrin	miR-6817-3p	NHERF2
miR-146a-3p	NHERF2	miR-3921	NHERF2	miR-6818-3p	NHERF2
miR-149-5p	Ezrin	miR-423-5p	NHERF1	miR-6818-5p	Ezrin
miR-155-5p	NHERF2	miR-4294	Ezrin	miR-6827-5p	NHERF2
miR-183-5p	Ezrin	miR-433-3p	CFTR	miR-6870-5p	NHERF2
miR-184	Ezrin	miR-4522	NHERF2	miR-6873-3p	NHERF2
miR-186-5p	Ezrin	miR-4526	Ezrin	miR-6888-3p	NHERF2
miR-18a-5p	Ezrin	miR-4653-5p	NHERF2	miR-6895-3p	NHERF2
miR-204-5p	Ezrin	miR-4711-3p	NHERF2	miR-7110-3p	NHERF2
miR-205-5p	Ezrin	miR-4723-5p	NHERF2	miR-7111-5p	NHERF2
miR-222-3p	Ezrin	miR-4766-5p	Ezrin	miR-877-3p	NHERF1
miR-223-3p	CFTR	miR-494-3p	CFTR	miR-92a-3p	Ezrin
miR-25-3p	Ezrin	miR-509-3p	CFTR	miR-939-5p	NHERF2
miR-301a-5p	NHERF2				

From: Chou et al. [5] and Huang et al. [6]. The involvement of NHERF1, NHERF2 and ezrin on CFTR expression can be found in Sharma et al., 2016 [7], Holcomb et al., 2014 [8] and Abbattiscianni, 2016 [9].

References

1. Giraldez, M.D.; Chevillet, J.R.; Tewari, M. Droplet Digital PCR for Absolute Quantification of Extracellular MicroRNAs in Plasma and Serum: Quantification of the Cancer Biomarker hsa-miR-141. *Methods Mol. Biol.* **2018**, *768*, 459-474.

2. Gasparello, J.; Papi, C.; Allegretti, M.; Giordani, E.; Carboni, F.; Zazza, S.; Pescarmona, E.; Romania, P.; Giacomini, P.; Scapoli, C.; Gambari, R.; Finotti, A. A Distinctive microRNA (miRNA) Signature in the Blood of Colorectal Cancer (CRC) Patients at Surgery. *Cancers (Basel).* **2020**, *12*, E2410.

3. Brognara, E.; Fabbri, E.; Bazzoli, E.; Montagner, G.; Ghimenton, C., Eccher, A.; Cantù, C.; Manicardi, A.; Bianchi, N.; Finotti, A.; Breveglieri, G.; Borgatti, M.; Corradini, R.; Bezzerri, V.; Cabrini, G.; Gambari, R. Uptake by human glioma cell lines and biological effects of a peptide-nucleic acids targeting miR-221. *J. Neurooncol.* **2014**, *118*, 19-28.

4. Fabbri, E.; Tamanini, A.; Jakova, T.; Gasparello, J.; Manicardi, A.; Corradini, R.; Finotti, A.; Borgatti, M.; Lampronti, I.; Munari, S.; Dechecchi, M.C.; Cabrini, G.; Gambari, R. Treatment of Calu-3 cells with a Peptide-Nucleic Acid (PNA) targeting the microRNA miR-101-3p is associated with increased expression of the Cystic Fibrosis Transmembrane Conductance Regulator (CFTR) gene. *Eur J Med Chem*, 2020, in press.

5. Chou, C.H.; Shrestha, S.; Yang, C.D.; Chang, N.W.; Lin, Y.L.; Liao, K.W.; Huang, W.C.; Sun, T.H.; Tu, S.J.; Lee, W.H.; Chiew, M.Y.; Tai, C.S.; Wei, T.Y.; Tsai, T.R.; Huang, H.T.; Wang, C.Y.; Wu, H.Y.; Ho, S.Y.; Chen, P.R.; Chuang, C.H.; Hsieh, P.J.; Wu, Y.S.; Chen, W.L.; Li, M.J.; Wu, J.C.; Huang, Y.C.; Ng, F.L.; Buddhakosai, W.; Huang, P.C.; Lan, K.C.; Huang, C.Y.; Weng, S.L.; Cheng, Y.N.; Liang, C.; Hsu, W.L.; Huang, H.D. miRTarBase update 2018: a resource for experimentally validated microRNA-target interactions. *Nucleic Acids Res.* **2017**, *46*, D296-D302.

6. Huang, H.Y.; Lin, Y.C.; Li, J.; Huang, K.Y.; Shrestha, S.; Hong, H.C.; Tang, Y.; Chen, Y.G.; Jin, C.N.; Yu, Y.; Xu, J.T.; Li, Y.M.; Cai, X.X.; Zhou, Z.Y.; Chen, X.H.; Pei, Y.Y.; Hu, L.; Su, J.J.; Cui, S.D.; Wang, F.; Xie, Y.Y.; Ding, S.Y.; Luo, M.F.; Chou, C.H.; Chang, N.W.; Chen, K.W.; Cheng, Y.H.; Wan, X.H.; Hsu, W.L.; Lee, T.Y.; Wei, F.X.; Huang, H.D. miRTarBase 2020: updates to the experimentally validated microRNA-target interaction database. *Nucleic Acids Res.* **2020**, *48*, D148-D154.

7. Sharma, N.; LaRusch, J.; Sosnay, P.R.; Gottschalk, L.B.; Lopez, A.P.; Pellicore, M.J.; Evans, T.; Davis, E.; Atalar, M.; Na, C.H.; Rosson, G.D.; Belchis, D.; Milewski, M.; Pandey, A.; Cutting, G.R.; A sequence upstream of canonical PDZ-binding motif within CFTR COOH-terminus enhances NHERF1 interaction. *Am. J. Physiol. Lung Cell. Mol. Physiol.* 2016, *311*, L1170-L1182.

8. Holcomb, J.; Jiang, Y.; Lu, G.; Trescott, L.; Brunzelle, J.; Sirinupong, N.; Li, C.; Naren, A.P.; Yang, Z. Structural insights into PDZ-mediated interaction of NHERF2 and LPA(2), a cellular event implicated in CFTR channel regulation. *Biochem Biophys Res Commun*. **2014**, *446*, 399-403.

9. Abbattiscianni, A.C.; Favia, M.; Mancini, M.T.; Cardone, R.A.; Guerra, L.; Monterisi, S.; Castellani, S.; Laselva, O.; Di Sole, F.; Conese, M.; Zaccolo, M.; Casavola, V. Correctors of mutant CFTR enhance subcortical cAMP-PKA signaling through modulating ezrin phosphorylation and cytoskeleton organization. *J. Cell Sci.* **2016**, *129*, 1128-1140.

© 2021 by the authors. Licensee MDPI, Basel, Switzerland. This article is an open access article distributed under the terms and conditions of the Creative Commons Attribution (CC BY) license (http://creativecommons.org/licenses/by/4.0/).
